# Long non-coding RNAs as novel prognostic biomarkers for breast cancer in Egyptian women

**DOI:** 10.1038/s41598-022-23938-8

**Published:** 2022-11-14

**Authors:** Basma El-Helkan, Manal Emam, Marwa Mohanad, Shadia Fathy, Abdel Rahman Zekri, Ola S. Ahmed

**Affiliations:** 1 Department of Biochemistry, Faculty of Science-Ain Shams University, Cairo, Egypt; 2 Virology and Immunology Unit, Cancer Biology Department, National Cancer Institute, Cairo University, Cairo, Egypt; 3 College of Pharmaceutical Sciences and Drug Manufacturing, Misr University for Science and Technology, 6th of October ,Giza, Egypt

**Keywords:** Cancer, Medical research

## Abstract

Breast cancer (BC), the most common type of malignant tumor, is the leading cause of death, having the highest incidence rate among women. The lack of early diagnostic tools is one of the clinical obstacles for BC treatment. The current study was designed to evaluate a panel of long non-coding RNAs **(**lncRNAs**)** BC040587, HOTAIR, MALAT1, CCAT1, CCAT2, PVT1, UCA1, SPRY4-IT1, PANDAR, and AK058003—and two mRNAs (SNCG, BDNF) as novel prognostic biomarkers for BC. This study was ethically approved by the Institutional Review Board of the National Cancer Institute, Cairo University. Our study included 75 women recently diagnosed with BC and 25 healthy women as normal controls. Patients were divided into three groups: 24 with benign breast diseases, 28 with metastatic breast cancer (MBC, stage IV), and 23 with non-metastatic breast cancer (NMBC, stage III). LncRNA and mRNA expression levels were measured in patient plasma using quantitative real-time PCR. We found that 10 lncRNAs (BCO40587, HOTAIR, PVT1, CCAT2, PANDAR, CCAT1, UCA1, SPRY4-IT1, AK058003, and MALAT1) and both mRNAs demonstrated at least a 2-fold change in expression with a more than 95% probability of significance. BCO40587 and SNCG were significantly up-regulated in MBC and NMBC patients (3.2- and 4-fold, respectively) compared with normal controls. The expression of UCA1 was repressed by 1.78-fold in MBC and NMBC patients compared with those with benign diseases. SPRY4-IT1 was down-regulated by 1.45-fold in MBC patients compared with NMBC and benign disease patients. Up-regulation of lncRNAs plays an important role in BC development. SNCG and BCO40587 may be potential prognostic markers for BC.

The organization number is IORG0003381 (IRB No: IRB00004025).

## Introduction

Breast cancer (BC) is a malignant tumor that has a high mortality rate and mostly occurs in women^1^. Although treatment strategies for molecular subtypes of BC have significantly progressed, the therapeutic response is often unsatisfactory^1^. Histopathological examination of tumor biopsies, which is an invasive procedure, remains the gold standard for diagnosing BC^2,3^. Other screening methods like mammography and breast examination were less effective in women with early breast tumors, aggressive tumors, and dense breast tissues^2,3^. BC metastasis is the leading cause of death, accounting for more than 90% of cancer-related deaths. Hence, new effective diagnostic and prognostic biomarkers are urgently needed to improve the survival rate and treatment efficiency of patients with BC^4^.

Genome-wide transcriptomics has revealed that protein-coding genes constitute only 2% of the human genome^5^_._ About 78% of the genome is composed of non-coding RNA (ncRNA) transcripts^5^. ncRNAs are divided into infrastructural and regulatory ncRNAs^6,7^. Infrastructural ncRNAs include ribosomal, transfer, small nuclear, and small nucleolar RNAs^6,7^. Regulatory ncRNAs include long ncRNAs (lncRNAs), microRNAs, piwi-interacting RNAs, and circular RNAs^6,7^.

LncRNAs are the longest members of this group, with sequences exceeding 200 nucleotides in length^8,9^. Most lncRNAs do not have open reading frames and coding potential, but they are transcribed by RNA polymerase II and undergo posttranscriptional modifications like coding RNAs^8,9^. LncRNAs are widespread in humans and are essential for regulating gene expression in physiological and pathological processes^10^. Advanced RNA sequencing and microarray technologies have shown that several lncRNAs play critical roles in BC progression by enhancing cancer cell proliferation, migration, and metastatic potential^11,12^. Thus, these molecules can be considered as promising diagnostic and therapeutic biomarkers for various cancers^13,14^. For example, overexpression of the lncRNAHox transcript antisense RNA (HOTAIR) was associated with an increased tumor aggressiveness and poor prognosis in both primary and metastatic breast tumors^15^.

The oncogenic lncRNA metastasis-associated lung adenocarcinoma transcript 1 (MALAT1) promoted triple-negative breast cancer proliferation and metastasis by regulating the expression of cell cycle genes^16^. Suppressing MALAT1 suppressed cancer cell proliferation by promoting cell cycle arrest in the G_2_/M phase and apoptosis^17^. Colon cancer associated transcript 2 (CCAT2) also served as a prognostic biomarker to predict the outcome of BC patients^18^.

LncRNAs affected the expression levels of some mRNAs such as The γ-synuclein gene (SNCG) and Brain-derived neurotrophic factor (BDNF). SNCG is a small naturally unfolded protein belonging to the synuclein family^19^. Normally, SNCG is expressed in peripheral neurons, ocular tissue, and adipose^20^. The expression of SNCG has been closely associated with tumor invasion and metastasis in a number of cancer types in a stage specific manner^21^. The expression of Ak058003 promotes the breast cancer proliferation, invasion and metastasis by regulating SNCG expression^22^.

BDNF is a member of the neurotrophin super-family which has been indicated in the pathophysiology of the nervous system as it is widely considered as a neuronal transcriptional factor regulating various aspects of neurogenesis and neuron growth in the human nervous system^23^. Over-expression of BDNF and TrkB has been demonstrated to act as a predictor of a poor clinical outcome and a worse survival when patients are suffering from human bladder cancer, neuroblastoma and breast carcinoma ^24–26^. Thus, assessing the expression of multiple lncRNAs as well as related genes, such as SNCG and BDNF, is imperative to better understand their roles in BC progression.

In this study, we investigated the expression profiles of a panel of plasma lncRNAs—BCO40584, AK58003, HOTAIR, CCAT1, CCAT2, MALAT1, PVT1, promoter of CDKN1A antisense DNA damage activated RNA (PANDAR), urothelial cancer associated 1 (UCA1), and sprouty RTK signaling antagonist 4-intronic transcript 1 (SPRY4-IT1) and the genes SNCG and BDNF in metastatic and non-metastatic BC cases compared with their expressions in benign breast diseases and healthy controls to identify the lncRNAs specifically associated with BC. We then explored whether the differentially expressed lncRNAs affected the expression of SNCG and BDNF. Furthermore, we examined the association between abnormal lncRNA expression and clinical pathology parameters of BC patients. We also calculate the correlation between LncRNAs & mRNAs expression levels and survival analysis of MBC & NMBC patients.

## Methods and materials

### Study subjects

This study included 51 BC patients diagnosed and treated at the National Cancer Institute, Cairo University, Egypt in the period from December 2017 to May 2020. Of them, 28 were metastatic cases (stage IV) and 23 were non-metastatic (stage III). The mean age of patients at the time of diagnosis was 49.8 ± 9.4 years for metastatic BC (MBC) and 52.6 ± 11.3 years for non-metastatic BC (NMBC). We also included 24 age-matched individuals with benign lesions and 25 apparently healthy normal controls. The mean ages of patients with benign lesions and healthy controls were 45.4 ± 8.4 and 47.96 ± 10.4 years, respectively. The clinical records of each patient were checked for characteristics including age, tumor size, pathologic staging, grading, lymph node status, and the expression of immunohistochemically confirmed hormone receptor markers: estrogen receptor (ER), progesterone receptor (PR), and human epidermal growth factor receptor 2 (HER2), as shown in Table 1. MBC patients were significantly associated with large tumor size (*P* = 0.003) and positive lymph node metastasis (*P* < 0.001). The study was approved by the National Cancer Institute’s Institutional Review Board and conducted in accordance with the 2013 Declaration of Helsinki. Written informed consent was obtained from each patient.

**Table 1:** Demographics of study participants and clinicopathological characteristics of metastatic and non-metastatic breast cancer patients.

Characteristics	MBC	NMBC	Benign breast lesions	Healthy Control	*P* value
	n = 28	n = 23	n = 24	n = 25	
Age (yrs)	49.8 ± 9.4	52.6 ± 11.3	45.4 ± 8.4	47.96 ± 10.4	0.22
**Age (yrs)**					
< 50	12 (42.9)	9 (39.13)	17 (70.9)	14 (56.0)	0.16
≥ 50	16 (57.1)	14 (60.87	7 (29.1)	11 (44.0)	
**Menopause**					
Pre	11 (39.3)	10 (43.48)	19 (79.2)	14 (56.0)	0.07
Post	17 (60.7)	13 (56.52)	5 (12.8)	11 (44.0)	
**Family history**					
No	22 (78.6)	18 (78.3)	21 (87.5)	–	1
Yes	6 (21.4)	5 (21.7)	3 (12.5)	–	
**Location**					
Right	13 (46.4)	16 (69.6)	11 (45.8)	–	
Left	14 (50.0)	7 (30.4)	12 (50.0)	–	0.42
Bilateral	1 (3.6)	0 (0.0)	1 (4.2)	–	
T. size (cm)	2.9 ± 1.46	1.8 ± 0.64	–	–	
**T. size (cm)**					
< 2.5	8 (28.6)	19 (82.6)	–	–	0.00036*
≥ 2.5	20 (71.4)	4 (17.4)	–	–	
**Type**					
DCIS	4 (14.29)	2 (8.69)			
IDC	22 (78.57)	19 (82.61)	–	–	
ILC	2 (7.14)	2 (8.69)	–	–	
**Grade**					
I	0 (0.0)	0 (15.8)	–	–	0.06
II	17 (60.7)	16 (69.57)	–	–	
III	11 (39.3)	7 (30.43)	–	–	
**Stage**					
3A	0 (0.00)	17 (73.9)	–	–	
3C	0 (0.00)	6 (26.1)	–	–	< 0.001*
4	28 (100.00)	0 (0.00)	–	–	
**LN metastasis**					
Negative	0 (0.0)	15 (65.22)	–		
Positive	28 (100.0)	8 (34.78)	–		< 0.001*
**ER**					
Negative	4 (14.3)	5 (21.7)	–	–	
Positive	24 (85.7)	18 (78.3)	–	–	0.82
**PR**					
Negative	4 (14.3)	4 (17.4)	–	–	
Positive	24 (85.7)	19 (82.6)	–	–	0.88
**Her2**					
Negative	20 (71.4)	16 (69.6)	–	–	0.99
Positive	8 (28.6)	7 (30.4)	–	–	
**Chemotherapy**					
No	16 (57.1)	14 (60.9)	–	–	1
Yes	12 (42.9)	9 (39.1)	–	–	
**Mortality**					
Alive	12 (42.9)	14 (60.8)	–	–	0.256
Dead	16 (57.1)	9 (39.2)	–	–	

MBC; metastatic breast cancer, NMBC, non-metastatic breast cancer, B, benign breast disease, NC; normal control, T. size; tumor size; DCIS; ductal carcinoma in situ, IDC; invasive ductal carcinoma, ILC; invasive lobular carcinoma, LN; lymph node, ER; estrogen receptor, PR; progesterone receptor, HER2; human epidermal growth factor receptor2.

### Sample collection

By direct venous puncture, 5 ml of blood samples were collected from subjects in tubes containing ethylenediaminetetraacetic acid and centrifuged at 1000 g for 5 min. The plasma samples were carefully transferred into RNase-free tubes for extraction of RNA, aliquoted, and stored at − 80 °C.

### RNA extraction and reverse transcription

RNA was extracted using the RNeasy Mini Kit for total RNA (Qiagen, GmBH, Cat. No. 74104), according to the manufacturer’s protocol. The concentration and quality of the extracted RNA were assessed using the NanoDrop 2000 spectrophotometer (Thermo Scientific, USA). The integrity of RNA was verified by the Agilent Bioanalyzer 2100 (Agilent Technologies, Inc.). Reverse transcription of 2 µg of the extracted RNA was carried out using the High-Capacity c-DNA Reverse Transcription Kit (Applied Biosystem, ThermoFisher Scientific, USA, Cat. No. k1691), according to the manufacturer’s protocol. Reverse transcription was performed in a 20 µl reaction volume at 25 °C for 10 min, 37 °C for 120 min, and finally, heat inactivation at 85 °C for 5 min.

### Quantitative real-time polymerase chain reaction (qRT-PCR)

qRT-PCR was performed in a 20 µl reaction volume using Maxima SYBR Green RT-PCR Kit (Thermo Scientific, USA, Cat. No. k0221) and the ABI 7900HT Fast Real-Time PCR system software. Each reaction included 20 ng of cDNA and 10 pmol of primers for HOTAIR, MALAT1, CCAT1, CCAT2, UCA1, PVT1, BC040587, SPRY4-IT1, PANDAR, AK058003, SNCG, BDNF, β-actin, and 18S rRNA. β-actin and 18S rRNA were used as housekeeping genes for normalization, as their expression levels are relatively stable in the plasma. The primers of lncRNAs, mRNAs and housekeeping genes were mentioned in Table 2. The thermal cycling protocol was as follows: an initial activation step for 10 min at 95 °C followed by 40 cycles of denaturation at 94 °C for 15 s, annealing at individually determined temperatures for 30 s, and DNA synthesis at 72 °C for 30 s. All reactions were performed in duplicates and melting curves were analyzed. Fold changes in mRNA levels were calculated using the 2^−ΔΔCt^ method. The ΔCts were obtained from the Cts normalized to those of the housekeeping genes.

**Table 2:** The primers of long noncoding RNAs, mRNAs, B- actin and 18srRNA.

Gene	Forward primer	Reverse primer	Tm
HOTAIR	5′TCATGATGGAATTGGAGCCTT-3′	5′CTCTTCCTGGCTTGCAGATTG-3′	57
MALAT1	5′CTTCCCTAGGGGATTTCAGG-3′	5′GCCCACAGGAACAAGTCCTA-3.′	55
UCA1	5′GCTTAATCCAGGAGACAAAG-3′	5′CATAGGTGTGAGTGGCG-3′	55
CCAT1	5’TCACTGACAACATCGACTTTGAAG-3’	5’GGAGAAAACGCTTAGCCATACAG-3’	60
CCAT2	5’CCCTGGTCAAATTGCTTAACCT-3’	5’TTATTCGTCCCTCTGTTTTATGGAT-3	57
PVT1	5′ATAGATCCTGCCCTGTTTGC-3′	5′CATTTCCTGCTGCCGTTTTC-3′	57
SPRY4-IT1	5′-ATCCGAAGCGCAGACACAATTCA-3′	5′CCTCGATGTAGTCTATGTCATAGGA-3′	57
SNCG	5'-CACCCTCTGGTCCTTCTG-3'	5'AGGAGTGGGCTCAAGTTT-3'	54
AK058003	5'CAGATGGCTGAGGTGGAAGG-3'	5'GACAAGGTCTCGCTCTTTTGCT-3'	55
BDNF	5’-TGG CTG ACA CTT TCG AAC AC-3’	5’-CCT CAT GGA CAT GTT TGC AG-3’)	52
PANDAR	TGCACACATTTAACCCGAAG	CCCCAAAGCTACATCTATGACA	55
BC040587	5′TAACAAGATTCACCTGCCAACC 3′	5′TGAGATCCAGAGTGTGCTGAAA 3′	57
18S rRNA	5′AGGATCCATTGGAGGGCAAGT-3′	5′TCCAACTACGAGCTTTTTAACTGCA-3′	55
B- actin	5′AGCACAGAGCCTCGCCTT-3′	CATCATCCATGGTGAGCTGG	60

### Statistical analysis

All statistical tests were performed using Studio software (version 3.6). The Shapiro–Wilk test was used to investigate the normality of the data. Different studied groups were compared with an individual’s clinicopathological characteristics using Pearson’s chi-squared test. Differential expression of lncRNAs among the investigated groups was compared with patients’ clinicopathological characteristics using the Kruskal–Wallis test with post-hoc Dunn’s test and Mann–Whitney U test. Hierarchical clustering was used to classify lncRNAs according to differential expression. The lncRNA expression correlation matrices were calculated using Spearman correlation. Overall survival (OS) analysis was performed using Kaplan–Meier curves and verified with the log-rank test estimator. The Cox regression hazard proportional model was applied for univariate and multivariate survival analysis. Statistical significance was indicated with a two-tailed *P* value of ≤ 0.05.

### Ethics approval and consent to participate

The study was ethically approved by the Institutional Review Boards (IRB) of the National Cancer Institute, Cairo University. Organization No·IORG0003381 (IRB NO·IRB00004025).

## Results

### Expression of lncRNAs and mRNAs

**Figure 1:**
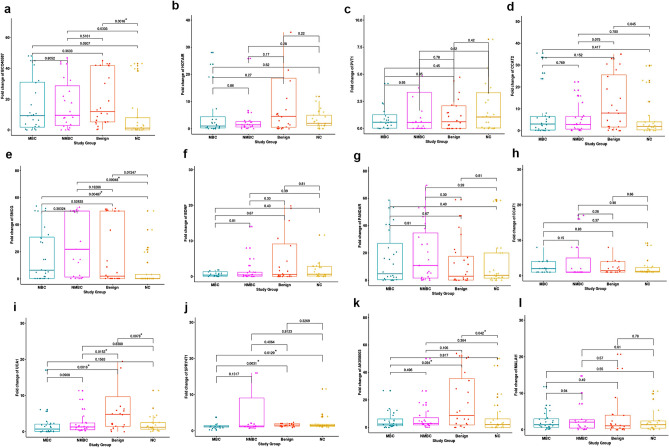
Differential expression of long non-coding RNAs (BCO40587, HOTAIR, PVT1, CCAT2, PANDAR, CCAT1, UCA1, SPRY4-IT1, AK058003 and MALAT1) and mRNAs (SNCG and BDNF) in different studied groups. *P* value refers to Mann–Whitney U test.

We investigated the fold change (FC) in expression of ten lncRNAs**(**BCO40587, HOTAIR, PVT1, CCAT2, PANDAR, CCAT1, UCA1, SPRY4-IT1, AK058003, and MALAT1) and two mRNAs (SNCG and BDNF) in patients with MBC, NMBC, benign disease, and normal controls (NC, Fig. 1, Supplementary 1)*.* The median FC in BCO40587 expression was elevated in MBC (9.32), NMBC (9.51) and benign diseases (11.9) compared with NC (1.23) (*P* = 0.05, *P* = 0.03, and *P* = 0.0016, respectively), which indicates that BC040587 is a good marker for all stages of BC. The median FC in UCA1 levels was higher for benign diseases (4.8) than for MBC (0.73), NMBC (1.3), and NC (1.24) (*P* = 0.0016, *P* = 0.0153, and *P* = 0.0078, respectively), which indicates that UCA1 is a good marker for BC. The median FC in SPRY4-IT1 expression was lower in MBC (1.09) compared with benign diseases (1.59) and NC (1.44) (*P* = 0.0031 and *P* = 0.013, respectively) with no difference compared with NMBC (median = 1.32, *P* = 0.406 and *P* = 0.81, respectively). These results indicate that SPRY4-IT1 is a good marker for MBC. The median FC in AK058003 expression was higher in benign breast diseases (8.71) than in MBC (2.4) and NC (2.0) (*P* = 0.034 and *P* = 0.042) with no difference when compared with NMBC (median = 2.9, *P* = 0.106). These results indicate that AK058003 is a good marker for MBC and benign breast diseases. The expressions of the other lncRNAs did not significantly differ between the different groups. The median FC in SNCG levels was higher in MBC (6.43) and NMBC (21.86) than in NC (0.26) (*P* = 0.0048 and *P* = 0.00049, respectively). However, no difference was found between SNCG expression in BC cases and patients with benign diseases, which indicate that SNCG is a good marker for MBC and NMBC.

### Associations between mRNAs and/or lncRNAs expression and clinicopathological features of MBC and NMBC patients

**Figure 2:**
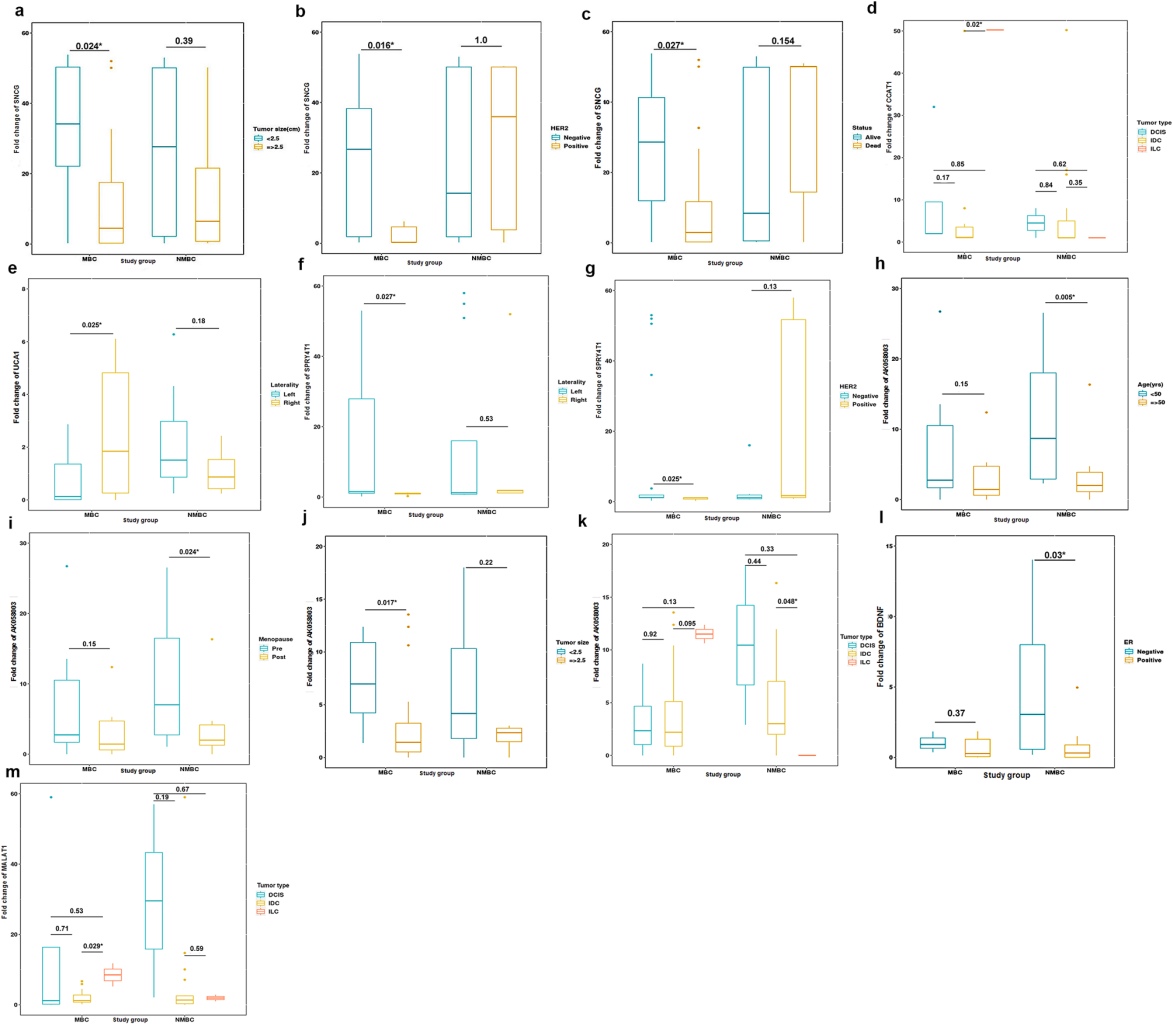
Clinicopathological features were significantly associated with lncRNAs/ mRNAs (**a–c**) SNCG with tumor size, HER2 overexpression and live status, (**d**) CCAT1 with tumor type, (**e**) UCA1with laterality, (**f, g**) SPRY4T*1* with laterality and HER2 overexpression, (**h–k**) AK058003 with age, menopause, tumor size and type, (**l**) BDNF with ER expression and (**m**) MALAT1 with tumor type.

We investigated the associations between the expressions of the studied lncRNAs and mRNAs and the clinicopathological characteristics of MBC and NMBC patients (Supplementary 2, Table 1). Reduced SNCG expression was significantly associated with tumor size (*P* = 0.024), HER2 overexpression (*P* = 0.016), and BC-related mortality in MBC patients (*P* = 0.027). However, SNCG expression was not related significantly with the clinicopathological features of NMBC patients. BDNF expression was significantly associated with negative ER expression in NMBC patients (*P* = 0.03)**.** Left breast tumors in MBC patients were significantly associated with UCA1 and SPRY4-IT1 down-regulation (*P* = 0.025 and *P* = 0.027, respectively). The median FC in SPRY4-IT1 expression was significantly reduced with HER2 overexpression in MBC patients (*P* = 0.025). The expression of AK058003 was significantly higher in MBC patients with tumor size of < 2.5 cm than in those with tumors ≥ 2.5 cm (*P* = 0.017). In NMBC patients, an up-regulation of AK058003 was significantly associated with young age (*P* = 0.005), premenopausal status (*P* = 0.024), and invasive ductal carcinoma compared with invasive lobular carcinoma (*P* = 0.048). Similarly, CCAT1 expression was significantly higher in MBC patients with ductal than lobular carcinomas (*P* = 0.02) (Fig. 2). These results indicate that SNCG, SPRY4IT1, AK058003 & CCAT1 expressions were associated with clinicopathological features of MBC patients. BDNF & AK058003 expressions were associated with clinicopathological features of NMBC patients.

### Correlations between expression of the studied lncRNAs&mRNAs in different patient cohorts

**Figure 3:**
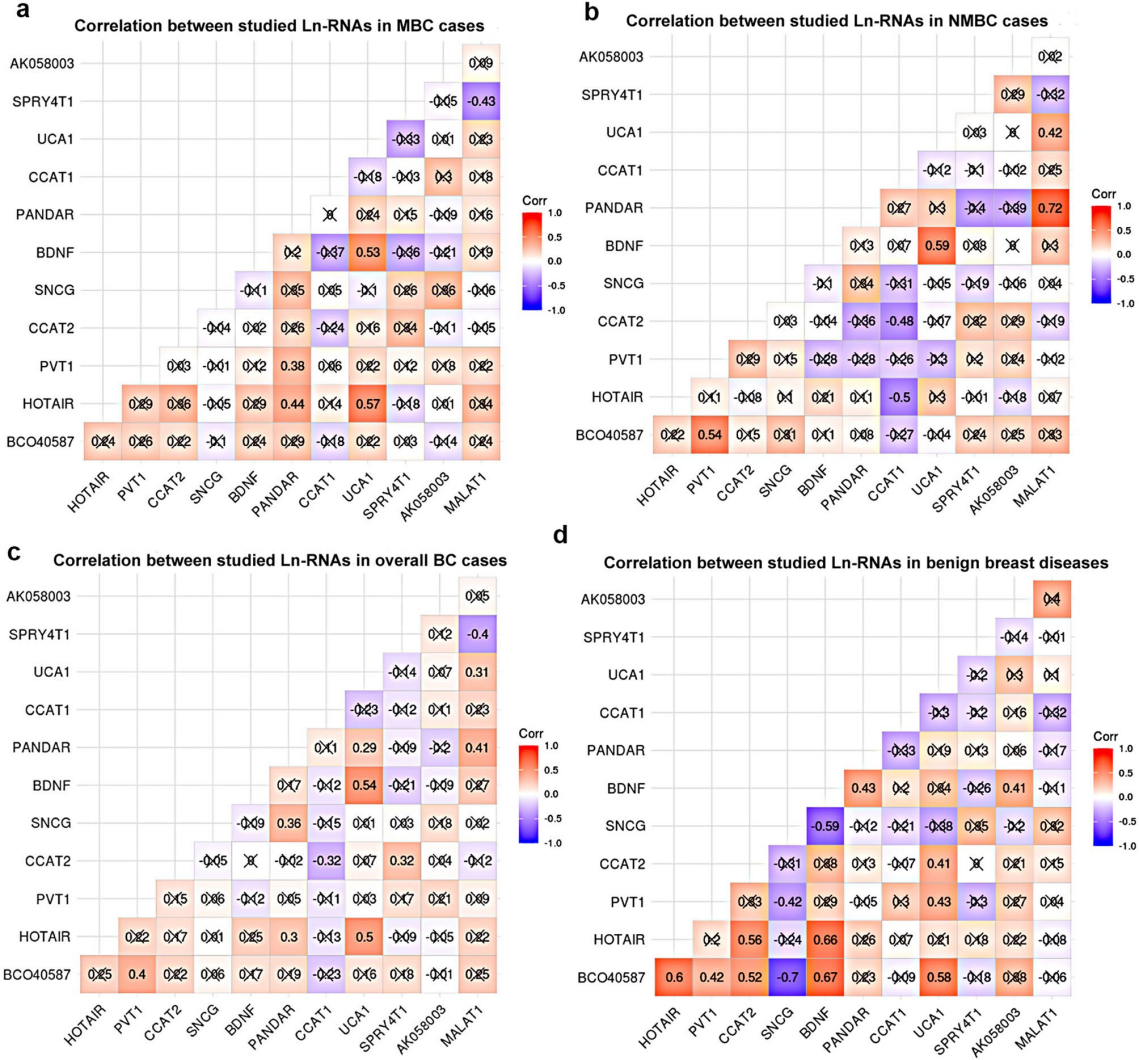
The correlation matrix shows spearman correlation coefficient between the expressions of studied long non-coding RNAs in (**a**) metastatic breast cancer, (**b**) non-metastatic breast cancer, (**c**) overall breast cancer cases and (**d**) benign breast diseases.

Using Spearman’s correlation, we evaluated the correlations between the expression levels of the studied lncRNAs (Fig. 3). In the overall BC cohort, BCO40587 expression was positively correlated with PVT1 expression (r = 0.40, *P* = 0.0036). UCA1 expression was positively correlated with the expressions of BDNF (r = 0.54, *P* < 0.0001), HOTAIR (r = 0.50, *P* = 0.00019), MALAT1 (r = 0.31, *P* = 0.025), and PANDAR (r = 0.29, *P* = 0.036). The expression of MALAT1 was negatively correlated with that of SPRY4-IT1 (r = 0.4, *P* = 0.0040) but positively correlated with that of PANDAR (r = 0.41, *P* = 0.0027). In MBC patients, UCA1 expression was positively correlated with BDNF (r = 0.53, *P* = 0.0036) and HOTAIR (r = 0.57, *P* = 0.0016) expression. The expression of PANDAR was positively correlated with HOTAIR (r = 0.44, *P* = 0.019) and PVT1 (r = 0.38, *P* = 0.043) expression. By contrast, the expressions of SPRY4-IT1 and MALAT1 were significantly inversely correlated (r = –0.43, *P* = 0.02). In NMBC patients, a moderate positive correlation was found between the expressions of BCO40587 and PVT1 (r = 0.54, *P* = 0.0076). UCA1 expression moderately positively correlated with BDNF (r = 0.594, *P* = 0.003) and MALAT1 (r = 0.42, *P* = 0.047) expression. A strong positive correlation was found between the expressions of MALAT1 and PANDAR (r = 0.72, *P* = 0.00012). By contrast, CCAT1 expression was moderately inversely correlated with the expressions of CCAT2 (r = –0.50, *P* = 0.014) and HOTAIR (r = –0.48, *P* = 0.019). In patients with benign breast disease, BCO40587 expression was positively correlated with BDNF (r = 0.67, *P* = 0.0003), HOTAIR *(*r = 0.6*, P* = *0.0019),* CCAT2 (r = 0.52, *P* = 0.009), and PVT1 (r = 0.42, *P* = 0.44) expressions. Moreover, UCA1 expression was positively correlated with BCO40587 (r = 0.58, *P* = 0.0029), PVT1 (r = 0.43, *P* = 0.037), and CCAT2 (r = 0.41, *P* = 0.044) expressions. A positive correlation was found between the expression of AK058003 and BDNF (r = 0.41, *P* = 0.048) as well as between HOTAIR expression and those of BDNF (r = 0.66, *P* = 0.0004) and CCAT2 (r = 0.56, *P* = 0.004). SNCG expression was inversely correlated with BDNF (r = − 0.59, *P* = 0.0025), BCO40587 (r = − 0.7, *P* = 0.00015), and PVT1 (r = − 0.42, *P* = 0.039) expressions.

### Survival analysis

**Figure 4:**
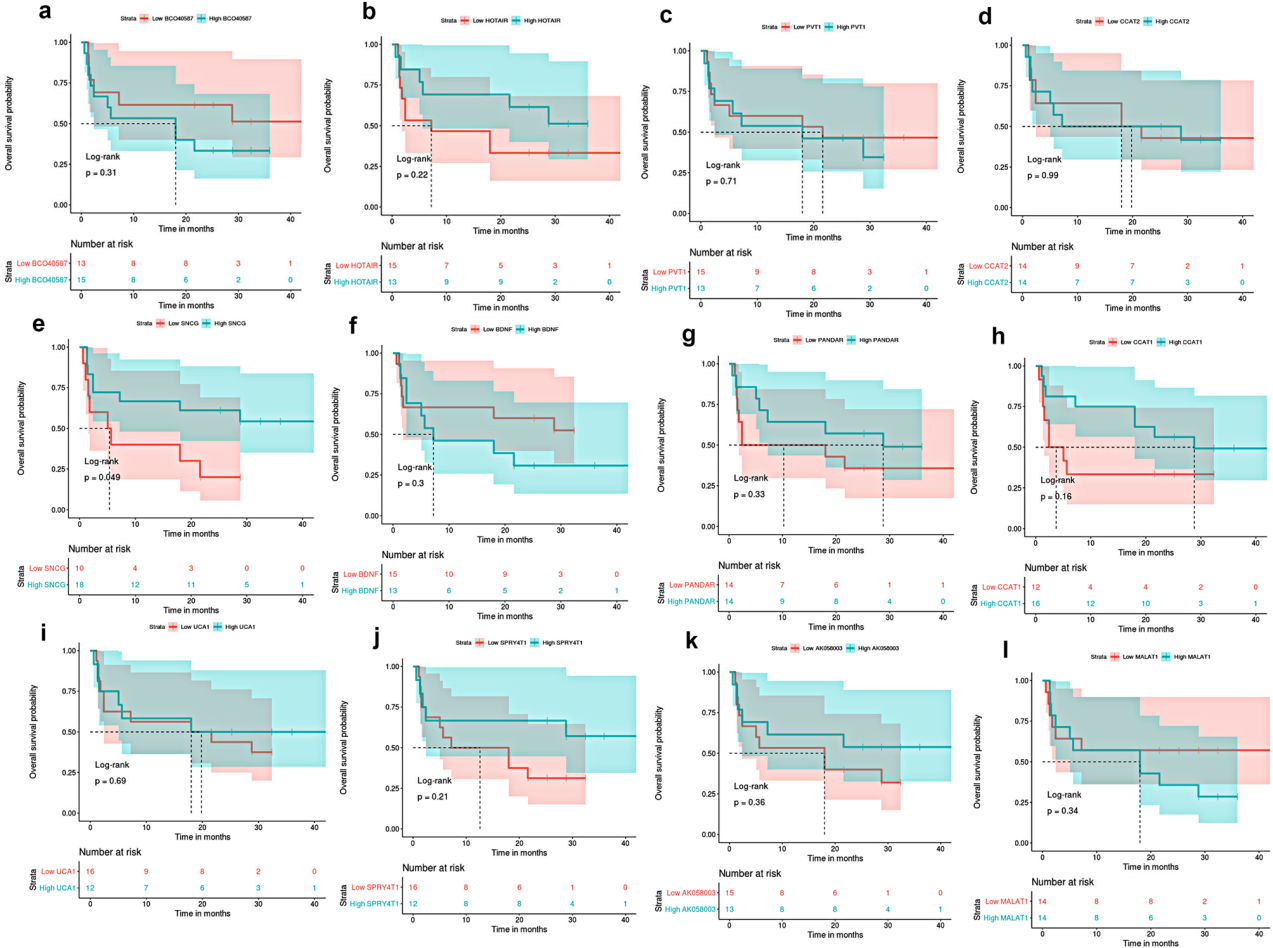
Kaplan Meier overall survival analysis of metastatic breast cancer patients in relation to lncRNAs (BCO40587, HOTAIR, PVT1, CCAT2, PANDAR, CCAT1, UCA1, SPRY4-IT1, AK058003 and MALAT1) and mRNAs (SNCG and BDNF) expression levels.

**Figure 5:**
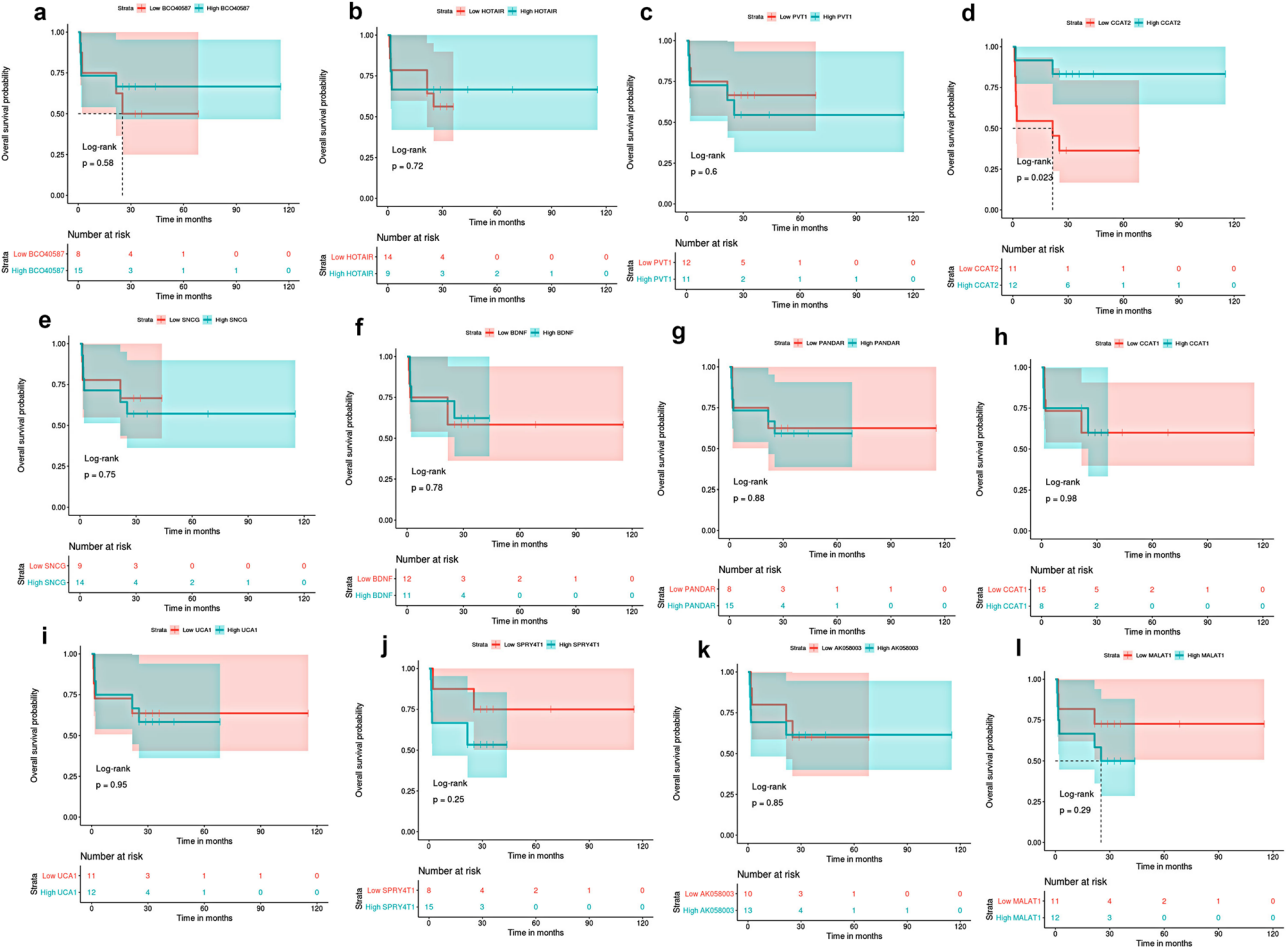
Kaplan Meier overall survival analysis of non-metastatic breast cancer patients in relation to lncRNAs (BCO40587, HOTAIR, PVT1, CCAT2, PANDAR, CCAT1, UCA1, SPRY4-IT1, AK058003 and MALAT1) and mRNAs (SNCG and BDNF) expression levels.

The median OS was 25.2 months (range, 0.6–115.2 months). Kaplan–Meier survival analysis showed that the OS rate did not differ between MBC and NMBC patients (*P* = 0.22, log-rank) **(**Fig. 1). Based on the median FC in expression, patients were categorized into two groups: those with high expression (≥ median FC) and those with low expression (< median FC). Then, Kaplan–Meier survival analysis examined the survival outcomes of MBC and NMBC patients associated with their lncRNA expressions. In MBC patients, a low expression of SNCG was associated with reduced OS (*P* = 0.049, log-rank) (Fig. 4). A low expression of CCAT2 was associated with decreased OS (*P* = 0.023, log-rank) in NMBC patients (Fig. 5). No relation was found between expressions of the other lncRNAs and patients’ outcomes. Univariate Cox regression analysis revealed that large tumor size, high tumor grade, HER2 overexpression, and a low expression of SNCG were associated with an increased risk of poor survival outcomes in MBC patients. HER2 overexpression and down-regulation of CCAT2 were associated with decreased OS in NMBC patients (Table 3). Multivariate survival analysis revealed that high tumor grade and low expression of SNCG were predictors of poorer survival outcomes in MBC patients, while low expression of CCAT2 was the only predictor of poor prognosis in NMBC patients.

**Table 3:** Univariate and multivariate overall survival analysis of MBC and NMBC patients.

Factors	Overall survival of MBC			Overall survival of NMBC		
	HR	95% CI	*P* value	HR	95% CI	*P* value
**Univariate**						
Age < 50 vs ≥ 50	2.10	0.75–5.90	0.10	2.3	0.48–11.1	0.30
T.size < 2.5 vs ≥ 2.5	4.94	1.11–22.02	0.01*	0.47	0.06–3.8	0.40
Grade 1–2 vs 3	8.57	2.91–25.19	< 0.0001*	0.60	0.12–2.88	0.50
Laterality L vs R	0.62	0.22–1.66	0.30	1.49	0.37–5.96	0.60
ER N vs P	0.24	0.06–0.91	0.04*	0.98	0.21–4.77	1.0
PR N vs P	0.20	0.02–1.76	0.10	2.07	0.26–16.56	0.50
HER2 N vs P	7.12	2.20–23.14	0.001*	4.02	0.99–16.23	0.04*
*BCO40587* L vs H	1.69	0.61–4.66	0.30	0.69	0.18–2.57	0.60
*HOTAIR* L vs H	0.52	0.19–1.45	0.20	0.77	0.82–1.1	0.70
*PVT1* L vs H	1.2	0.45–3.20	0.70	1.42	0.38–5.29	0.60
*CCAT2* L vs H	0.99	0.37–2.64	1.0	0.19	0.04–0.94	0.02*
*SNCG* L vs H	0.38	0.14–1.04	0.048*	1.25	0.31–5.01	0.70
*BDNF* L vs H	1.69	0.62–4.61	0.30	0.82	0.22–3.05	0.80
*PANDAR* L vs H	0.61	0.22–1.65	0.30	1.10	0.28–4.41	0.90
*CCAT1* L vs H	0.49	0.18–1.33	0.20	1.01	0.25–4.04	1.0
*UCA1* L vs H	0.81	0.29–0.23	0.70	1.05	0.28–3.89	0.90
*SPRY4T1* L vs H	0.51	0.17–1.49	0.20	2.47	0.51–11.93	0.20
*AK058003* L vs H	0.62	0.22–1.72	0.40	1.10	0.30–4.22	0.90
*MALAT1* L vs H	1.63	0.59–4.49	0.30	2.08	0.52–8.33	0.30
**Multivariate**						
T.size < 2.5 vs ≥ 2.5	0.93	0.15–5.76	0.94			
Grade 1–2 vs 3	12.5	2.79–56.37	< 0.0001*			
HER2 N vs P	2.77	0.82–9.42	0.11	3.67	0.90–15.03	0.07
SNCG L vs H	0.21	0.06–0.74	0.01*			
*CCAT2* L vs H				0.21	0.04–1.03	0.048*

HR; hazard ratio, CI; confidence interval, MBC; metastatic breast cancer, NMBC; non-metastatic breast cancer, T. size, tumor size, ER; estrogen receptor, PR; progesterone receptor, HER2; human epidermal growth factor receptor2.

## Discussion

BC is the second most common type of cancer with high morbidity and mortality rates^27^^.^LncRNAs can regulate different aspects of gene expression, including chromatin organization, transcriptional regulation, and posttranscriptional control^28^. Dysregulation of lncRNA expression has been linked to cancer and other human disorders^28^. Many lncRNAs play oncogenic roles in BC, such as HOTAIR, MALAT1, UCA1, PANDAR, CCAT1, CCAT2, SPRY4-IT1, and AK058003^29^. Some lncRNAs act as tumor suppressors in breast cancer, such as BC040587 and growth arrest specific 5^29^.

In the current study, MALAT1 was down-regulated but SNCG and HOTAIR were significantly up-regulated in patients with benign breast diseases. These results agree with previous studies that have suggested that MALAT1 acts as a tumor suppressor gene^30^, and that HOTAIR was significantly up-regulated in breast tumors^31^. In contrast, Wu et al*.* found that 81% of stage III/IV BCs were positive for SNCG expression, but this proportion was only 15% for stage I/II BCs and SNCG was undetectable in benign breast lesions^32^.

In the present study, SNCG, BC040587, CCAT2, UCA1, AK058003, MALAT1, and PANDAR were up-regulated in NMBC patients. These results corroborate previous studies that have reported the up-regulation of all these lncRNAs in BC tissues^33–42^. In contrast, Chi et al*.*^43^, found that the expression of BC040587 declined in breast cancer tissues compared with normal tissues. Recent studies have reported a suppressive role for MALAT-1 in BC^30,44–46^.

In the present study, BDNF, HOTAIR, PVT1, CCAT1, and SPRY4-IT1 were down-regulated in NMBC patients. Huth et al*.* have reported the suppressive properties of BDNF in BC development^47^. However, our results contrast those of previous studies, wherein the levels of all these lncRNAs have been shown to be elevated in BC tissues^48–53^.

In patients with MBC, UCA1 was found to be down-regulated, which is contrary to Li et al*.* who reported that the expression of AC026904.1 and UCA1 was higher in MBC than in NMBC^35^. Also, we found that SNCG expression was lower in MBC patients than in NMBC patients, but Wu et al*.* have reported that the relative SNCG mRNA levels were higher in the MBC group in their study^32^. In our study, AK058003 levels were lower in MBC compared with NMBC patients, while a previous study has demonstrated the opposite trend^33^. Our findings about CCAT2 expression corroborate those of Wu et al., CCAT2 was up-regulated in MBC compared with NMBC patients^34^.

The present study showed that the expressions of BC040587, HOTAIR, PVT1, and CCAT2 were not correlated with clinicopathological features in MBC and NMBC patients. These results agree with Abd El-Fattah et al., who reported that PVT1 was not significantly associated with other clinicopathological features^49^. Another study demonstrated that BC040587 was significantly correlated with menopausal status but its expression in breast cancer was not associated with other parameters such as age, tumor location, tumor size, ER status, PR status, P53 status, Ki67 status, lymph node status, and TNM staging^43^. HOTAIR expression has been shown to be associated with lymph node metastases but not with tumor size or grading^28^. Tan et al*.* reported that high CCAT2 expression was significantly associated with cancer growth and metastasis, including tumor size, clinical stage, and TNM classification but other factors like age, gender, and histological differentiation were not significant^46^. In contrast to our results, studies have reported a significant association between serum HOTAIR expression, tumor size, and tumor stage, over-expression of HOTAIR in HER2-positive samples than in negative ones, and a significant association between PVT1 expression, tumor size, and TNM stage of breast cancer^49,55,56^.

The current study showed that CCAT1 expression was significantly associated with tumor type in MBC patients, but was not correlated with any clinicopathological features in NMBC patients. However, a previous study has reported CCAT1 expression to be significantly correlated with differentiation grade, TNM stage, and lymph node metastases in BC patients, but not with other factors, such as age, tumor size, ER, PR, and HER2 status^57^.

We showed that PANDAR expression was significantly associated with family history in MBC patients, but no other correlations were established with clinicopathological features of NMBC patients. Previously, PANDAR expression was shown to be positively associated with lymph node metastasis and advanced clinical stage in patients^42^.

In the present study, UCA1 expression was significantly associated with laterality in MBC patients, while this lncRNA has been shown to be significantly associated with tumor size and stage of BC, previously^35^.

SPRY4-IT1 expression was significantly associated with laterality and HER2 in MBC patients. A previous study showed that an up-regulation of SPRY4-IT1 was associated with larger tumor size and later stage of tumor development in BC patients^53^.

We found MALAT1 expression to be significantly associated with tumor type in MBC patients. Recent studies have showed that high MALAT1 expression was associated with increased tumor stage, recurrence, decreased survival, lymph node size, ER expression, and histological grade^41,58^.

In our study, AK058003 expression was related to age, menopausal status, and tumor type in NMBC patients, while it was associated with tumor size in MBC patients. A previous study has also shown AK058003 expression to be associated with the extent of lymph node metastasis^33^.

We found that BDNF expression was associated with ER status in NMBC but it did not correlate with any clinicopathological features in MBC patients. Higher BDNF levels have been shown to be associated with unfavorable pathological parameters, including nodal positivity, and adverse clinical outcomes, including local recurrence, death, poor prognosis, and reduced disease-free survival and OS. The expression of BDNF did not increase with the TNM stage^48^.

In the present study, SNCG expression was significantly associated with tumor type, HER2, and live status in MBC patients. However, a previous study reported that SNCG expression in the primary tumor was also significantly associated with lymph node involvement and metastasis^32^. No significant correlation was established between SNCG gene expression, age, menstruation, and the status of ER, PR, proliferating cell nuclear antigen, and HER2.

We demonstrated that AK058003 was positively correlated with BDNF expression, while this lncRNA was positively correlated with SNCG expression in a previous study^33^.

In our study, low SNCG expression was significantly associated with reduced OS in MBC patients (p = 0.049, log-rank), while Wu et al. reported that SNCG-positive patients showed a significantly poorer prognosis than SNCG-negative patients^32^. Low CCAT2 expression was significantly associated with decreased OS (p = 0.023, log-rank) in NMBC patients, while Tan et al. found that CCAT2 was significantly correlated with OS and progression-free survival^54^.

## Conclusion

Our study clearly demonstrates that the identification of reliable lncRNA biomarkers for BC demands multiple validation studies in independent patient cohorts with large sample sizes that take into account tumor stages and molecular subtypes as well. In addition, lncRNAs might warrant investigation as components of biomarker panels for BC.

## Supplementary Information

Supplementary Information 1.

Supplementary Information 2.

## Data Availability

The datasets used and analysed during the current study available from the corresponding author on reasonable request.

## Abbreviations

BC: Breast cancer
NC: Healthy women as normal control
BB: Benign breast tumor
MBC: Metastatic breast cancer
NMBC: Non-metastatic breast cancer
OS: Overall survival
