# Supplementary material for: Long non-coding RNAs as novel prognostic biomarkers for breast cancer in Egyptian women
Source: Sci Rep. 2022 Nov 14;12:19498. doi: 10.1038/s41598-022-23938-8 (PMC9663553; doi:10.1038/s41598-022-23938-8)
Supplement: Supplementary file 1 — Supplementary Information 1. [file 41598_2022_23938_MOESM1_ESM.docx]

**Table S1. Fold change expression of long non-coding RNA among different studied Groups**

| **Characteristics** | **MBC** | **NMBC** | **B** | **NC** | **P^a^-value** |  | **P^b^-value** |
| --- | --- | --- | --- | --- | --- | --- | --- |
| **BCO40587** |  |  |  |  | 0.019* | MBC vs NMBC | 0.80 |
| Mean ± SD | 16.28±18.02 | 16.75±16.3 | 19.7±17.05 | 9.9±16.3 |  | MBC vs B | 0.363 |
| 95% CI | 9.2-23.3 | 9.8-23.7 | 12.7-26.7 | 3.18-16.54 |  | MBC vs NC | 0.05* |
| Median | 9.32 | 9.51 | 11.9 | 1.23 |  | NMBC vs B | 0.516 |
| IQR | 29.23 | 25.7 | 36.46 | 7.91 |  | NMBC vs NC | 0.0303* |
|  |  |  |  |  |  | B vs NC | 0.0016* |
| **HOTAIR** |  |  |  |  | 0.41 | MBC vs NMBC | 0.86 |
| Mean ± SD | 11.27±22.7 | 5.74±14.98 | 15.2±22.89 | 3.28±3.25 |  | MBC vs B | 0.27 |
| 95% CI | 2.46-20.08 | -0.67-12.1 | 5.66-24.8 | 1.95-4.61 |  | MBC vs NC | 0.52 |
| Median | 1.017 | 1.46 | 4.58 | 2.01 |  | NMBC vs B | 0.17 |
| IQR | 4.13 | 2.02 | 18.06 | 3.81 |  | NMBC vs NC | 0.28 |
|  |  |  |  |  |  | B vs NC | 0.22 |
| **PVT1** |  |  |  |  |  | MBC vs NMBC | 0.95 |
| Mean ± SD | 6.18±18.1 | 11.3±24.7 | 4.02±14.12 | 7.05±18.9 | 0.85 | MBC vs B | 0.85 |
| 95% CI | -0.84-13.2 | 0.71-21.9 | -1.9-9.9 | -0.70-14.8 |  | MBC vs NC | 0.45 |
| Median | 0.57 | 0.55 | 0.62 | 1.06 |  | NMBC vs B | 0.78 |
| IQR | 1.24 | 3.33 | 2.09 | 3.25 |  | NMBC vs NC | 0.62 |
|  |  |  |  |  |  | B vs NC | 0.42 |
| **CCAT2** |  |  |  |  |  | MBC vs NMBC | 0.769 |
| Mean ± SD | 7.65±11.28 | 5.51±6.76 | 12.6±12.90 | 6.09±9.48 |  | MBC vs B | 0.152 |
| 95% CI | 3.27-12.03 | 2.61-8.40 | 7.19-18.00 | 2.19-9.98 | 0.169 | MBC vs NC | 0.417 |
| Median | 3.04 | 2.80 | 8.05 | 1.90 |  | NMBC vs B | 0.075 |
| IQR | 6.28 | 5.83 | 23.89 | 3.66 |  | NMBC vs NC | 0.78 |
|  |  |  |  |  |  | B vs NC | 0.19 |
| **SNCG** |  |  |  |  |  | MBC vs NMBC | 0.303 |
| Mean ± SD | 17.97±19.73 | 26.15±22.90 | 17.75±22.96 | 6.96±13.20 |  | MBC vs B | 0.53 |
| 95% CI | 10.32-25.62 | 16.35-35.94 | 8.12-27.38 | 1.54-12.38 | 0.0047* | MBC vs NC | 0.0048* |
| Median | 6.43 | 21.86 | 2.04 | 0.26 |  | NMBC vs B | 0.182 |
| IQR | 30.6 | 48.7 | 49.84 | 3.04 |  | NMBC vs NC | 0.00049* |
|  |  |  |  |  |  | B vs NC | 0.072 |
| **BDNF** |  |  |  |  |  | MBC vs NMBC | 0.81 |
| Mean ± SD | 5.99±13.76 | 1.66±3.30 | 6.79±11.56 | 1.71±2.46 |  | MBC vs B | 0.67 |
| 95% CI | 0.66-11.33 | 0.25-3.07 | -3.19-6.50 | 0.70-2.71 | 0.72 | MBC vs NC | 0.40 |
| Median | 0.39 | 0.45 | 0.59 | 0.65 |  | NMBC vs B | 0.30 |
| IQR | 1.32 | 1.13 | 9.13 | 2.71 |  | NMBC vs NC | 0.39 |
|  |  |  |  |  |  | B vs NC | 0.81 |
| **PANDAR** |  |  |  |  |  | MBC vs NMBC | 0.81 |
| Mean ± SD | 16.29±19.61 | 19.86±21.29 | 12.16±16.89 | 14.54±20.93 |  | MBC vs B | 0.67 |
| 95% CI | 8.68-23.89 | 10.75-28.97 | 5.09-19.24 | 5.96-23.13 |  | MBC vs NC | 0.40 |
| Median | 4.74 | 10.78 | 2.95 | 3.6 | 0.48 | NMBC vs B | 0.30 |
| IQR | 24.60 | 33.08 | 17.45 | 18.4 |  | NMBC vs NC | 0.39 |
|  |  |  |  |  |  | B vs NC | 0.81 |
| **CCAT1** |  |  |  |  |  | MBC vs NMBC | 0.15 |
| Mean ± SD | 8.31±15.88 | 5.49±10.84 | 7.37±14.71 | 4.04±9.90 |  | MBC vs B | 0.83 |
| 95% CI | 2.15-14.47 | 0.85-10.13 | 1.21-13.53 | -0.02-8.10 | 0.48 | MBC vs NC | 0.37 |
| Median | 2.0 | 1.0 | 1.44 | 1.23 |  | NMBC vs B | 0.28 |
| Range | 3.0 | 4.0 | 3.0 | 1.30 |  | NMBC vs NC | 0.40 |
|  |  |  |  |  |  | B vs NC | 0.66 |
| **UCA1** |  |  |  |  |  | MBC vs NMBC | 0.09 |
| Mean ± SD | 3.24±8.32 | 2.30±2.86 | 10.37±14.32 | 2.09±2.51 |  | MBC vs B | 0.0016* |
| 95% CI | 0.01-6.46 | 1.08-3.53 | 4.38-16.37 | 1.06-3.12 | 0.0030* | MBC vs NC | 0.156 |
| Median | 0.73 | 1.30 | 4.80 | 1.24 |  | NMBC vs B | 0.0153* |
| IQR | 2.04 | 1.88 | 8.19 | 1.85 |  | NMBC vs NC | 0.838 |
|  |  |  |  |  |  | B vs NC | 0.0078* |
| **SPRY4T1** |  |  |  |  |  | MBC vs NMBC | 0.132 |
| Mean ± SD | 7.82±16.88 | 11.70±20.31 | 8.22±18.05 | 2.21±2.21 |  | MBC vs B | 0.0031* |
| 95% CI | 1.28-14.37 | 3.02-20.39 | 0.66-15.78 | 1.30-3.11 | 0.02* | MBC vs NC | 0.0129* |
| Median | 1.09 | 1.32 | 1.59 | 1.44 |  | 0.406 |  |
| Range | 0.39 | 7.98 | 0.71 | 0.56 |  | NMBC vs NC | 0.81 |
|  |  |  |  |  |  | B vs NC | 0.326 |
| **AK058003** |  |  |  |  |  | MBC vs NMBC | 0.496 |
| Mean ± SD | 4.86±6.04 | 7.44±11.44 | 18.95±20.59 | 8.0±13.72 |  | MBC vs B | 0.034* |
| 95% CI | 2.51-7.19 | 2.55-12.34 | 10.33-27.58 | 2.37-13.63 | 0.096 | MBC vs NC | 0.817 |
| Median | 2.40 | 2.90 | 8.71 | 2.0 |  | NMBC vs B | 0.106 |
| IQR | 4.99 | 5.22 | 33.77 | 6.19 |  | NMBC vs NC | 0.364 |
|  |  |  |  |  |  | B vs NC | 0.042* |
| **MALAT1** |  |  |  |  |  | MBC vs NMBC | 0.94 |
| Mean ± SD | 4.31±11.02 | 7.38±16.36 | 5.42±12.62 | 4.58±11.88 |  | MBC vs B | 0.49 |
| 95% CI | 0.04-8.59 | 0.38-14.38 | 0.14-10.71 | -0.30-9.45 |  | MBC vs NC | 0.55 |
| Median | 1.37 | 2.08 | 1.07 | 1.38 | 0.85 | NMBC vs B | 0.57 |
| Range | 2.32 | 2.28 | 3.71 | 1.96 |  | NMBC vs NC | 0.61 |
|  |  |  |  |  |  | B vs NC | 0.78 |

^a^Kruskal Wallis was used to compare between the four groups. ^b^Pairwise comparison was tested with Mann Whitney with adjusted Post hoc Dunn’s test.
