# Supplementary material for: Long non-coding RNAs as novel prognostic biomarkers for breast cancer in Egyptian women
Source: Sci Rep. 2022 Nov 14;12:19498. doi: 10.1038/s41598-022-23938-8 (PMC9663553; doi:10.1038/s41598-022-23938-8)
Supplement: Supplementary file 2 — Supplementary Information 2. [file 41598_2022_23938_MOESM2_ESM.docx]

**Table S2-A.** Association of *BCO40587* expression with clinicopathological characteristics of metastatic and non-metastatic breast cancer patients.

| **Features** | **MBC** |  | **p^a^** | **NMBC** |  | **p^a^** |
| --- | --- | --- | --- | --- | --- | --- |
|  | **Median (IQR)** |  |  | **Median (IQR)** |  |  |
| **Age** |  |  |  |  |  |  |
| <50 | 6.32(26.0) | <50 vs ≥ 50 |  | 8.28(23.7) | <50 vs ≥ 50 |  |
| ≥ 50 | 9.92(28.5) |  | 0.75 | 14.8(25.8) |  | 0.53 |
| **Menopause** |  |  |  |  |  |  |
| Pre | 6.32(26.0) | Pre vs Post | 0.75 | 8.1(18.9) | Pre vs Post | 0.38 |
| Post | 9.92(28.5) |  |  | 17.6(28.8) |  |  |
| **Tumor Grade** |  |  |  |  |  |  |
| 1-2 | 9.92(41.15) | 1-2 vs 3 | 0.59 | 14.8(28.0) | 1-2 vs 3 | 0.26 |
| 3 | 6.68(14.5) |  |  | 7.84(13.0) |  |  |
| **Laterality** |  |  |  |  |  |  |
| Left | 9.32(17.7) | Left vs Right |  | 8.14(16.5) | Left vs Right |  |
| Right | 6.89(40.3) |  | 0.85 | 32.2(37.0) |  | 0.20 |
| **Tumor size** |  |  |  |  |  |  |
| <2.5 | 5.8(2.1) | <2.5 vs ≥ 2.5 | 0.25 | 12.0(28.9) | <2.5 vs ≥ 2.5 | 0.18 |
| ≥ 2.5 | 9.6(39.7) |  |  | 4.25(10.4) |  |  |
| **Family History** |  |  |  |  |  |  |
| No | 6.5(9.97) | No vs Yes | 0.068 | 10.7(28.7) | No vs Yes | 0.65 |
| Yes | 36.5(26.6) |  |  | 8.28(13.9) |  |  |
| **Type** |  |  |  |  |  |  |
| DCIS | 5.8(17.5) | DCIS vs IDC | 0.72 | 25.4(17.6) | DCIS vs IDC | 0.47 |
| IDC | 9.6(29.0) | DCIS vs ILC | 1.0 | 8.28(18.5) | DCIS vs ILC | 0.67 |
| ILC | 5.6(5.6) | IDC vs ILC | 0.46 | 39.5(7.31) | IDC vs ILC | 0.06 |
| **Stage** |  |  |  |  |  |  |
| 3A | - |  |  | 9.51(16.8) | 3A vs 3C | 0.73 |
| 3C | - |  |  | 13.8(31.2) |  |  |
| 4 | 9.32(29.2) |  |  | - |  |  |
| **LN metastasis** |  |  |  |  |  |  |
| No | - |  |  | 8.28(14.6) | No vs Yes | 0.46 |
| Yes | 9.32(29.2) |  |  | 23.5(34.0) |  |  |
| **ER** |  |  |  |  |  |  |
| Negative | 3.5(1.97) | -ve vs +ve | 0.37 | 12.0(20.1) | -ve vs +ve | 0.85 |
| Positive | 9.92(31.3) |  |  | 8.9(27.7) |  |  |
| **PR** |  |  |  |  |  |  |
| Negative | 2.38(0.0) | -ve vs +ve | 0.53 | 17.3(21.8) | -ve vs +ve | 0.57 |
| Positive | 9.45(29.9) |  |  | 8.28(24.6) |  |  |
| **HER2** |  |  |  |  |  |  |
| Negative | 9.92(30.0) | -ve vs +ve | 0.94 | 8.0(16.2) | -ve vs +ve | 0.12 |
| Positive | 6.32(24.2) |  |  | 23.5(34.6) |  |  |
| **Live status** |  |  |  |  |  |  |
| Alive | 3.01(19.5) | A vs D | 0.20 | 8.9(12.9) | A vs D | 0.49 |
| Dead | 10.3(29.6) |  |  | 21.9(40.6) |  |  |

^a^Mann Whitney U testused forpairwise comparison. MBC; metastatic breast cancer, NMBC; non-metastatic breast cancer, IQR; interquartile range, LN; lymph node,ER; estrogen receptor, PR; progesterone receptor, HER2; human epidermal growth factor receptor2, DCIs; ductal carcinoma insitu, IDC; invasive ductal carcinoma, ILC; invasive lobular carcinoma, A; alive, D; dead.

**Table S2-B.** Association of *HOTAIR* expression with clinicopathological characteristics of metastatic and non-metastatic breast cancer patients.

| **Features** | **MBC** |  | **p^a^** | **NMBC** |  | **p^a^** |
| --- | --- | --- | --- | --- | --- | --- |
|  | **Median (IQR)** |  |  | **Median (IQR)** |  |  |
| **Age** |  |  |  |  |  |  |
| <50 | 1.55(5.15) | <50 vs ≥ 50 | 0.89 | 0.87(0.88) | <50 vs ≥ 50 | 0.21 |
| ≥ 50 | 0.99(2.93) |  |  | 1.92(1.80) |  |  |
| **Menopause** |  |  |  |  |  |  |
| Pre | 1.55(5.15) | Pre vs Post | 0.89 | 0.80(1.1) | Pre vs Post | 0.08 |
| Post | 0.99(2.93) |  |  | 2.33(1.94) |  |  |
| **Tumor Grade** |  |  |  |  |  |  |
| 1-2 | 2.22(18.6) | 1-2 vs 3 | 0.16 | 1.51(3.15) | 1-2 vs 3 | 0.48 |
| 3 | 0.73(1.21) |  |  | 1.0(1.17) |  |  |
| **Laterality** |  |  |  |  |  |  |
| Left | 0.52(1.96) | Left vs Right | 0.1 | 1.27(1.72) | Left vs Right | 1.0 |
| Right | 1.95(25.1) |  |  | 1.46(35.1) |  |  |
| **Tumor size** |  |  |  |  |  |  |
| <2.5 | 1.36(4.03) | <2.5 vs ≥ 2.5 | 0.9 | 1.46(2.04) | <2.5 vs ≥ 2.5 | 0.75 |
| ≥ 2.5 | 1.02(7.2) |  |  | 1.27(1.1) |  |  |
| **Family History** |  |  |  |  |  |  |
| No | 0.72(2.89) | No vs Yes | 0.14 | 1.51(2.03) | No vs Yes | 0.41 |
| Yes | 2.89(4.13) |  |  | 0.87(0.44) |  |  |
| **Type** |  |  |  |  |  |  |
| DCIS | 2.73(5.7) | DCIS vs IDC | 0.41 | 0.73(0.14) | DCIS vs IDC | 0.37 |
| IDC | 0.71(3.0) | DCIS vs ILC | 0.80 | 1.5(1.7) | DCIS vs ILC | 0.33 |
| ILC | 12.6(11.1) | IDC vs ILC | 0.32 | 6.3(0.35) | IDC vs ILC | 0.08 |
| **Stage** |  |  |  |  |  |  |
| 3A | - |  | NA | 1.03(1.63) | 3A vs 3C | 0.51 |
| 3C | - |  |  | 2.22(2.18) |  |  |
| 4 | 1.02(4.13) |  |  | - |  |  |

| **LN metastasis** |  |  |  |  |  |  |
| --- | --- | --- | --- | --- | --- | --- |
| No | - |  | NA | 1.03(1.71) | No vs Yes | 0.61 |
| Yes | 1.02(4.13) |  |  | 1.91(3.11) |  |  |
| **ER** |  |  |  |  |  |  |
| Negative | 0.74(0.41) | -ve vs +ve | 0.46 | 2.4(2.4) | -ve vs +ve | 0.63 |
| Positive | 1.55(7.01) |  |  | 1.25(1.60) |  |  |
| **PR** |  |  |  |  |  |  |
| Negative | 0.74(0.0) | -ve vs +ve | 0.90 | 0.79(6.6) | -ve vs +ve | 0.66 |
| Positive | 1.05(5.15) |  |  | 1.5(1.9) |  |  |
| **HER2** |  |  |  |  |  |  |
| Negative | 1.05(3.1) | -ve vs +ve | 0.83 | 1.52(1.52) | -ve vs +ve | 0.54 |
| Positive | 0.99(14.3) |  |  | 1.01(2.04) |  |  |
| **Livstatus** |  |  |  |  |  |  |
| Alive | 1.36(3.2) | A vs D | 0.87 | 1.01(2.2) | A vs D | 0.85 |
| Dead | 1.02(7.2) |  |  | 1.51(1.34) |  |  |

^a^ Mann Whitney U testused forpairwise comparison. MBC; metastatic breast cancer, NMBC; non-metastatic breast cancer, IQR; interquartile range, LN; lymph node,ER; estrogen receptor, PR; progesterone receptor, HER2; human epidermal growth factor receptor2, DCIs; ductal carcinoma insitu, IDC; invasive ductal carcinoma, ILC; invasive lobular carcinoma, A; alive, D; dead.

**Table S2-C.** Association of *PVT1* expression with clinicopathological characteristics of metastatic and non-metastatic breast cancer patients.

| **Features** | **MBC** |  | **p^a^** | **NMBC** |  | **p^a^** |
| --- | --- | --- | --- | --- | --- | --- |
|  | **Median (IQR)** |  |  | **Median (IQR)** |  |  |
| **Age** |  |  |  |  |  |  |
| <50 | 0.54(0.84) | <50 vs ≥ 50 | 0.73 | 0.22(0.63) | <50 vs ≥ 50 | 0.21 |
| ≥ 50 | 0.77(1.55 |  |  | 1.29(4.5) |  |  |
| **Menopause** |  |  |  |  |  |  |
| Pre | 0.54(0.84) | Pre vs Post | 0.73 | 0.22(0.61 | Pre vs Post | 0.17 |
| Post | 0.77(1.55 |  |  | 1.59(4.82) |  |  |
| **Tumor Grade** |  |  |  |  |  |  |
| 1-2 | 0.59(2.15) | 1-2 vs 3 | 0.48 | 0.82(4.0) | 1-2 vs 3 | 0.66 |
| 3 | 0.56(0.88) |  |  | 0.54(0.49) |  |  |
| **Laterality** |  |  |  |  |  |  |
| Left | 0.68(1.38) | Left vs Right | 0.70 | 0.59(9.5) | Left vs Right | 0.63 |
| Right | 0.50(0.91) |  |  | 0.54(2.26) |  |  |
| **Tumor size** |  |  |  |  |  |  |
| <2.5 | 1.22(5.2) | <2.5 vs ≥ 2.5 | 0.15 | 0.56(3.3) | <2.5 vs ≥ 2.5 | 0.77 |
| ≥ 2.5 | 0.51(0.81) |  |  | 0.50(7.4) |  |  |
| **Family History** |  |  |  |  |  |  |
| No | 0.51(1.1) | No vs Yes | 0.19 | 0.59(3.5) | No vs Yes | 0.73 |
| Yes | 0.82(11.4) |  |  | 0.54(0.64) |  |  |
| **Type** |  |  |  |  |  |  |
| DCIS | 0.73(0.54) | DCIS vs IDC | 0.43 | 0.59(0.05) | DCIS vs IDC | 0.95 |
| IDC | 0.5(1.05) | DCIS vs ILC | 0.27 | 0.56(3.3) | DCIS vs ILC | 1.0 |
| ILC | 1.7(0.66) | IDC vs ILC | 0.23 | 2.4(2.4) | IDC vs ILC | 1.0 |
| **Stage** |  |  |  |  |  |  |
| 3A |  |  |  | 0.64(3.73) | 3A vs 3C |  |
| 3C |  |  |  | 0.55(0.48) |  | 0.80 |
| 4 | 0.57(1.2) |  |  | - |  |  |
| **LN metastasis** |  |  |  | 0.56(2.6) |  | 0.82 |
| No |  |  |  | 0.32(19.2) | No vs Yes |  |
| Yes | 0.57(1.24) |  |  |  |  |  |
| **ER** |  |  |  |  |  |  |
| Negative | 0.59(0.24) | -ve vs +ve | 0.97 | 0.63(1.04) | -ve vs +ve | 0.30 |
| Positive | 0.56(1.55) |  |  | 0.38(3.5) |  |  |
| **PR** |  |  |  |  |  |  |
| Negative | 0.29(0.0) | -ve vs +ve | 0.71 | 0.55(0.12) | -ve vs +ve | 0.84 |
| Positive | 0.59(1.3) |  |  | 0.64(4.3) |  |  |
| **HER2** |  |  |  |  |  |  |
| Negative | 0.54(1.14) | -ve vs +ve | 0.41 | 0.64(3.7) | -ve vs +ve | 0.78 |
| Positive | 0.77(1.2) |  |  | 0.55(2.1) |  |  |
| **Live status** |  |  |  |  |  |  |
| Alive | 0.19(0.96) | A vs D | 0.23 | 0.38(1.35) | A vs D | 0.48 |
| Dead | 0.68(1.02) |  |  | 0.99(3.7) |  |  |

^a^ Mann Whitney U testused forpairwise comparison. MBC; metastatic breast cancer, NMBC; non-metastatic breast cancer, IQR; interquartile range, LN; lymph node,ER; estrogen receptor, PR; progesterone receptor, HER2; human epidermal growth factor receptor2, DCIs; ductal carcinoma insitu, IDC; invasive ductal carcinoma, ILC; invasive lobular carcinoma, A; alive, D; dead.

**Table S2-D.** Association of *CCAT2* expression with clinicopathological characteristics of metastatic and non-metastatic breast cancer patients.

| **Features** | **MBC** |  | **p^a^** | **NMBC** |  | **p^a^** |
| --- | --- | --- | --- | --- | --- | --- |
|  | **Median (IQR)** |  |  | **Median (IQR)** |  |  |
| **Age** |  |  |  |  |  |  |
| <50 | 2.53(4.88) | <50 vs ≥ 50 | 0.48 | 6.19(12.8) | <50 vs ≥ 50 | 0.14 |
| ≥ 50 | 5.28(6.43) |  |  | 1.10(3.93) |  |  |
| **Menopause** |  |  |  |  |  |  |
| Pre | 1.15(3.35) | Pre vs Post | 0.48 | 4.86(10.7) | Pre vs Post | 0.14 |
| Post | 2.44(4.86) |  |  | 0.91(4.57) |  |  |
| **Tumor Grade** |  |  |  |  |  |  |
| 1-2 | 2.35(7.7) | 1-2 vs 3 | 0.67 | 2.97(7.57) | 1-2 vs 3 | 0.66 |
| 3 | 4.26(4.79) |  |  | 1.29(5.78) |  |  |
| **Laterality** |  |  |  |  |  |  |
| Left | 1.65(6.4) | Left vs Right | 0.49 | 3.33(7.18) | Left vs Right | 0.061 |
| Right | 4.18(4.58) |  |  | 0.27(2.98) |  |  |
| **Tumor size** |  |  |  |  |  |  |
| <2.5 | 1.65(3.7) | <2.5 vs ≥ 2.5 | 0.29 | 2.81(5.28) | <2.5 vs ≥ 2.5 | 0.87 |
| ≥ 2.5 | 4.18(10.4) |  |  | 3.7(7.9) |  |  |
| **Family History** |  |  |  |  |  |  |
| No | 2.06(5.85) | No vs Yes | 0.12 | 2.97(5.48) | No vs Yes | 0.94 |
| Yes | 5.68(19.0) |  |  | 2.77(6.07) |  |  |
| **Type** |  |  |  |  |  |  |
| DCIS | 0.86(0.65) | DCIS vs IDC | 0.20 | 11.2(11.2) | DCIS vs IDC | 0.90 |
| IDC | 4.70(7.19) | DCIS vs ILC | 1.0 | 2.81(5.83) | DCIS vs ILC | 1.0 |
| ILC | 2.13(2.12) | IDC vs ILC | 0.30 | 2.99(2.18) | IDC vs ILC | 0.95 |
| **Stage** |  |  |  |  |  |  |
| 3A | - |  |  | 3.14(6.05) | 3A vs 3C | 0.31 |
| 3C | - |  |  | 0.95(2.81) |  |  |
| 4 | 3.04(6.22) |  |  | - |  |  |
| **LN metastasis** |  |  |  |  |  |  |
| No | - |  |  | 5.17(9.19) | No vs Yes | 0.48 |
| Yes | 3.04(6.22) |  |  | 1.84(2.56) |  |  |
| **ER** |  |  |  |  |  |  |
| Negative | 5.3(3.2) | -ve vs +ve | 0.94 | 2.81(2.24) | -ve vs +ve | 1.0 |
| Positive | 2.71(6.43) |  |  | 2.95(6.05) |  |  |
| **PR** |  |  |  |  |  |  |
| Negative | 6.4(0.0) | -ve vs +ve | 0.46 | 2.41(3.22) | -ve vs +ve | 0.74 |
| Positive | 2.71(6.16) |  |  | 2.81(9.19) |  |  |
| **HER2** |  |  |  |  |  |  |
| Negative | 2.53(5.76) | -ve vs +ve | 0.49 | 5.17(12.1) | -ve vs +ve | 0.34 |
| Positive | 5.28(12.9) |  |  | 2.03(2.94) |  |  |
| **Live status** |  |  |  |  |  |  |
| Alive | 2.95(5.61) | A vs D | 0.96 | 4.35(5.40) | A vs D | 0.30 |
| Dead | 3.49(6.33) |  |  | 0.91(2.50) |  |  |

^a^ Mann Whitney U testused forpairwise comparison. MBC; metastatic breast cancer, NMBC; non-metastatic breast cancer, IQR; interquartile range, LN; lymph node,ER; estrogen receptor, PR; progesterone receptor, HER2; human epidermal growth factor receptor2, DCIs; ductal carcinoma insitu, IDC; invasive ductal carcinoma, ILC; invasive lobular carcinoma, A; alive, D; dead.

**Table S2-E.** Association of *SNCG* expression with clinicopathological characteristics of metastatic and non-metastatic breast cancer patients.

|  | **MBC** |  | **p^a^** | **NMBC** |  | **p^a^** |
| --- | --- | --- | --- | --- | --- | --- |
|  | **Median (IQR)** |  |  | **Median (IQR)** |  |  |
| **Age** |  |  |  |  |  |  |
| <50 | 14.3(32.5) | <50 vs ≥ 50 | 0.56 | 0.91(49.8) | <50 vs ≥ 50 | 0.17 |
| ≥ 50 | 4.86(29.7) |  |  | 38.5(35.8) |  |  |
| **Menopause** |  |  |  |  |  |  |
| Pre | 14.3(32.5) | Pre vs Post | 0.56 | 1.36(42.7) | Pre vs Post | 0.18 |
| Post | 4.86(29.7) |  |  | 49.3(35.9) |  |  |
| **Tumor Grade** |  |  |  |  |  |  |
| 1-2 | 14.3(38.1) | 1-2 vs 3 | 0.49 | 24.8(48.0) | 1-2 vs 3 | 0.59 |
| 3 | 5.17(19.6) |  |  | 14.4(49.1) |  |  |
| **Laterality** |  |  |  |  |  |  |
| Left | 10.5(29.5) | Left vs Right | 0.63 | 18.1(49.3) | Left vs Right | 0.44 |
| Right | 5.17(29.3) |  |  | 50.1(41.8) |  |  |
| **Tumor size** |  |  |  |  |  |  |
| <2.5 | 34.2(28.2) | <2.5 vs ≥ 2.5 | **0.024*** | 27.7(47.9) | <2.5 vs ≥ 2.5 | 0.39 |
| ≥ 2.5 | 4.5(17.2) |  |  | 6.48(20.8) |  |  |
| **Family History** |  |  |  |  |  |  |
| No | 5.01(31.7) | No vs Yes | 0.44 | 18.1(49.2) | No vs Yes | 1.0 |
| Yes | 20.8(20.9) |  |  | 49.3(48.3) |  |  |
| **Type** |  |  |  |  |  |  |
| DCIS | 34.2(17.1) | DCIS vs IDC | 0.16 | 26.0(24.1) | DCIS vs IDC | 1.0 |
| IDC | 5.01(29.3) | DCIS vs ILC | 0.13 | 21.9(49.5) | DCIS vs ILC | 1.0 |
| ILC | 3.2(2.97) | IDC vs ILC | 0.52 | 32.2(17.9) | IDC vs ILC | 0.86 |
| **Stage** |  |  |  |  |  |  |
| 3A | - |  |  | 21.9(48.3) | 3A vs 3C | 0.97 |
| 3C | - |  |  | 32.3(46.4) |  |  |
| 4 | 6.44(30.6) |  |  |  |  |  |
| **LN metastasis** |  |  |  |  |  |  |
| No | - |  |  | 14.4(47.9) | No vs Yes | 1.0 |
| Yes | 6.44(30.6) |  |  | 38.9(49.7) |  |  |
| **ER** |  |  |  |  |  |  |
| Negative | 4.1(2.5) | -ve vs +ve | 0.28 | 14.4(19.3) | -ve vs +ve | 0.71 |
| Positive | 14.3(32.4) |  |  | 38.5(49.0) |  |  |
| **PR** |  |  |  |  |  |  |
| Negative | 0.26(0.0) | -ve vs +ve | 0.23 | 7.34(23.1) | -ve vs +ve | 0.22 |
| Positive | 6.68(30.5) |  |  | 27.7(48.0) |  |  |
| **HER2** |  |  |  |  |  |  |
| Negative | 26.7(36.5) | -ve vs +ve | **0.016*** | 14.2(48.3) | -ve vs +ve | 1.0 |
| Positive | 0.26(4.4) |  |  | 36.0(46.3) |  |  |
| **Live status** |  |  |  |  |  |  |
| Alive | 28.6(29.4) | A vs D | **0.027*** | 8.39(49.3)) | A vs D | 0.154 |
| Dead | 2.94(11.4) |  |  | 50.1(35.8) |  |  |

^a^ Mann Whitney U testused forpairwise comparison. MBC; metastatic breast cancer, NMBC; non-metastatic breast cancer, IQR; interquartile range, LN; lymph node, ER; estrogen receptor, PR; progesterone receptor, HER2; human epidermal growth factor receptor2, DCIs; ductal carcinoma insitu, IDC; invasive ductal carcinoma, ILC; invasive lobular carcinoma, A; alive, D; dead. *Significance at p<0.05.

**Table S2-F.** Association of *BDNF* expression with clinicopathological characteristics of metastatic and non-metastatic breast cancer patients.

| **Features** | **MBC** |  | **p^a^** | **NMBC** |  | **p^a^** |
| --- | --- | --- | --- | --- | --- | --- |
|  | **Median (IQR)** |  |  | **Median (IQR)** |  |  |
| **Age** |  |  |  |  |  |  |
| <50 | 0.10(0.78) | <50 vs ≥ 50 | 0.06 | 0.50(4.65) | <50 vs ≥ 50 | 0.19 |
| ≥ 50 | 0.90(1.64) |  |  | 0.26(0.90) |  |  |
| **Menopause** |  |  |  |  |  |  |
| Pre | 0.10(0.78) | Pre vs Post | 0.06 | 0.84(4.1) | Pre vs Post | 0.07 |
| Post | 0.90(1.64) |  |  | 0.19(0.57) |  |  |
| **Tumor Grade** |  |  |  |  |  |  |
| 1-2 | 0.27(1.76) | 1-2 vs 3 | 0.54 | 0.54(1.12) | 1-2 vs 3 | 0.11 |
| 3 | 0.41(0.86) |  |  | 0.19(0.38) |  |  |
| **Laterality** |  |  |  |  |  |  |
| Left | 0.35(0.85) | Left vs Right | 0.80 | 0.40(1.60) | Left vs Right | 0.87 |
| Right | 0.49(1.73) |  |  | 0.45(0.38) |  |  |
| **Tumor size** |  |  |  |  |  |  |
| <2.5 | 0.40(1.58) | <2.5 vs ≥ 2.5 | 0.82 | 0.45(1.2) | <2.5 vs ≥ 2.5 | 0.31 |
| ≥ 2.5 | 0.39(0.98) |  |  | 0.24(0.66) |  |  |
| **Family History** |  |  |  |  |  |  |
| No | 0.55(1.47) | No vs Yes | 0.59 | 0.39(1.39) | No vs Yes | 0.94 |
| Yes | 0.15(0.44) |  |  | 0.45(0.7) |  |  |
| **Type** |  |  |  |  |  |  |
| DCIS | 1.03(9.16) | DCIS vs IDC | 0.41 | 0.22(0.22) | DCIS vs IDC | 0.34 |
| IDC | 0.39(0.88) | DCIS vs ILC | 1.0 | 0.48(1.04) | DCIS vs ILC | 0.67 |
| ILC | 20.3(20.3) | IDC vs ILC | 0.96 | 0.76(0.75) | IDC vs ILC | 1.0 |
| **Stage** |  |  |  |  |  |  |
| 3A |  |  |  | 0.48(1.2) | 3A vs 3C | 0.67 |
| 3C |  |  |  | 0.38(5.9) |  |  |
| 4 | 0.39(1.32) |  | NA |  |  |  |
| **LN metastasis** |  |  |  |  |  |  |
| No |  |  |  | 0.45(0.79) | No vs Yes | 0.21 |
| Yes | 0.39(1.32) |  | NA | 0.77(2.9) |  |  |
| **ER** |  |  |  |  |  |  |
| Negative | 0.92(0.73) | -ve vs +ve | 0.37 | 3.1(7.4) | -ve vs +ve | **0.03*** |
| Positive | 0.28(1.24) |  |  | 0.31(0.89) |  |  |
| **PR** |  |  |  |  |  |  |
| Negative | 0.37(0.0) | -ve vs +ve | 1.0 | 2.7(6.8) | -ve vs +ve | 0.18 |
| Positive | 0.41(1.4) |  |  | 0.32(1.1) |  |  |
| **HER2** |  |  |  |  |  |  |
| Negative | 0.20(0.89) | -ve vs +ve | 0.14 | 0.18(0.58) | -ve vs +ve | 0.05 |
| Positive | 0.92(21.1) |  |  | 0.98(4.2) |  |  |
| **Livestatus** |  |  |  |  |  |  |
| Alive | 0.096(0.75) | A vs D | 0.17 | 0.48(1.13) | A vs D | 0.73 |
| Dead | 0.80(9.15) |  |  | 0.32(1.02) |  |  |

^a^ Mann Whitney U testused forpairwise comparison. MBC; metastatic breast cancer, NMBC; non-metastatic breast cancer, IQR; interquartile range, LN; lymph node, ER; estrogen receptor, PR; progesterone receptor, HER2; human epidermal growth factor receptor2, DCIs; ductal carcinoma insitu, IDC; invasive ductal carcinoma, ILC; invasive lobular carcinoma, A; alive, D; dead. *Significance at p<0.05.

**Table S2-G.** Association of *PANDAR* expression with clinicopathological characteristics of metastatic and non-metastatic breast cancer patients.

| **Features** | **MBC** |  | **p^a^** | **NMBC** |  | **p^a^** |
| --- | --- | --- | --- | --- | --- | --- |
|  | **Median (IQR)** |  |  | **Median (IQR)** |  |  |
| **Age** |  |  |  |  |  |  |
| <50 | 3.46(38.5) | <50 vs ≥ 50 | 0.69 | 5.39(18.6) | <50 vs ≥ 50 | 0.55 |
| ≥ 50 | 15.2(18.4) |  |  | 20.7(39.0) |  |  |
| **Menopause** |  |  |  |  |  |  |
| Pre | 3.46(38.5) | Pre vs Post | 0.69 | 8.08(22.0) | Pre vs Post | 1.0 |
| Post | 15.2(18.4) |  |  | 14.9(36.7) |  |  |
| **Tumor Grade** |  |  |  |  |  |  |
| 1-2 | 15.2(39.6) | 1-2 vs 3 | 0.26 | 10.6(36.2) | 1-2 vs 3 | 0.55 |
| 3 | 2.23(15.0) |  |  | 10.8(25.7) |  |  |
| **Laterality** |  |  |  |  |  |  |
| Left | 3.2(12.2) | Left vs Right | 0.36 | 5.3(28.4) | Left vs Right | 0.19 |
| Right | 21.3(43.1) |  |  | 20.7(23.6) |  |  |
| **Tumor size** |  |  |  |  |  |  |
| <2.5 | 10.6(40.6) | <2.5 vs ≥ 2.5 | 0.17 | 10.8(33.1) | <2.5 vs ≥ 2.5 | 0.94 |
| ≥ 2.5 | 2.59(21.2) |  |  | 15.9(33.4) |  |  |
| **Family History** |  |  |  |  |  |  |
| No | 2.8(17.8) | No vs Yes | **0.046*** | 8.6(28.9) | No vs Yes | 0.48 |
| Yes | 44.9(32.7) |  |  | 26.2(41.3) |  |  |
| **Type** |  |  |  |  |  |  |
| DCIS | 28.0(28.5) | DCIS vs IDC | 0.27 | 39.6(13.5) | DCIS vs IDC | 0.21 |
| IDC | 4.48(22.6) | DCIS vs ILC | 0.10 | 5.4(27.9) | DCIS vs ILC | 0.67 |
| ILC | 1.34(1.32) | IDC vs ILC | 0.32 | 28.1(13.2) | IDC vs ILC | 0.31 |
| **Stage** |  |  |  |  |  |  |
| 3A |  |  | NA | 14.9(36.6) | 3A vs 3C | 0.78 |
| 3C |  |  |  | 8.1(19.7) |  |  |
| 4 | 4.74(26.6) |  |  |  |  |  |
| **LN metastasis** |  |  |  |  |  |  |
| No |  |  | NA | 20.7(40.3) | No vs Yes | 0.50 |
| Yes | 4.74(26.6) |  | NA | 5.9(14.2) |  |  |
| **ER** |  |  |  |  |  |  |
| Negative | 2.17(9.2) | -ve vs +ve | 0.65 | 2.6(3.4) | -ve vs +ve | 0.41 |
| Positive | 6.02(36.4) |  |  | 17.8(34.1) |  |  |
| **PR** |  |  |  |  |  |  |
| Negative | 0.42(0.0) | -ve vs +ve | 0.46 | 3.30(16.4) | -ve vs +ve | 0.47 |
| Positive | 6.02(29.5) |  |  | 14.9(32.4) |  |  |
| **HER2** |  |  |  |  |  |  |
| Negative | 6.02(35.6) | -ve vs +ve | 0.79 | 14.9(23.9) | -ve vs +ve | 1.0 |
| Positive | 2.17(20.9) |  |  | 8.1(37.5) |  |  |
| **Live status** |  |  |  |  |  |  |
| Alive | 11.0(40.7) | A vs D | 0.43 | 10.2(24.9) | A vs D | 0.75 |
| Dead | 2.80(18.9) |  |  | 10.8(35.7) |  |  |

^a^ Mann Whitney U testused forpairwise comparison. MBC; metastatic breast cancer, NMBC; non-metastatic breast cancer, IQR; interquartile range, LN; lymph node, ER; estrogen receptor, PR; progesterone receptor, HER2; human epidermal growth factor receptor2, DCIs; ductal carcinoma insitu, IDC; invasive ductal carcinoma, ILC; invasive lobular carcinoma, A; alive, D; dead. *Significance at p<0.05.

**Table S2-H.** Association of *CCAT1* expression with clinicopathological characteristics of metastatic and non-metastatic breast cancer patients.

| **Features** | **MBC** |  | **p^a^** | **NMBC** |  | **p^a^** |
| --- | --- | --- | --- | --- | --- | --- |
|  | **Median (IQR)** |  |  | **Median (IQR)** |  |  |
| **Age** |  |  |  |  |  |  |
| <50 | 2.0(3.09) | <50 vs ≥ 50 | 0.34 | 1.0(1.8) | <50 vs ≥ 50 | 0.28 |
| ≥ 50 | 1.08(1.14) |  |  | 1.0(1.0) |  |  |
| **Menopause** |  |  |  |  |  |  |
| Pre | 2.0(3.09) | Pre vs Post | 0.34 | 1.0(7.0) | Pre vs Post | 0.12 |
| Post | 1.08(1.14) |  |  | 1.0(0.75) |  |  |
| **Tumor Grade** |  |  |  |  |  |  |
| 1-2 | 2.0(3.0) | 1-2 vs 3 | 0.61 | 1.0(2.5) | 1-2 vs 3 | 0.91 |
| 3 | 1.11(3.0) |  |  | 1.0(3.5) |  |  |
| **Laterality** |  |  |  |  |  |  |
| Left | 2.0(2.48) | Left vs Right | 0.91 | 1.0(1.75) | Left vs Right | 0.25 |
| Right | 1.81(3.0) |  |  | 2.0(4.0) |  |  |
| **Tumor size** |  |  |  |  |  |  |
| <2.5 | 5.0(34.8) | <2.5 vs ≥ 2.5 | 0.14 | 1.0(1.0) | <2.5 vs ≥ 2.5 | 0.32 |
| ≥ 2.5 | 1.37(1.61) |  |  | 8.5(15.3) |  |  |
| **Family History** |  |  |  |  |  |  |
| No | 1.81(2.54) | No vs Yes | 0.51 | 1.0(1.0) | No vs Yes | 0.69 |
| Yes | 3.0(23.8) |  |  | 1.0(7.0) |  |  |
| **Type** |  |  |  |  |  |  |
| DCIS | 2.0(2.75) | DCIS vs IDC | 0.17 | 4.5(3.5) | DCIS vs IDC | 0.84 |
| IDC | 1.09(2.54) | DCIS vs ILC | 0.085 | 1.0(4.0) | DCIS vs ILC | 0.62 |
| ILC | 50.2(0.05) | IDC vs ILC | **0.02*** | 1.0(0.0) | IDC vs ILC | 0.35 |
| **Stage** |  |  |  |  |  |  |
| 3A | - |  | NA | 1.0(1.0) | 3A vs 3C | 0.90 |
| 3C | - |  |  | 1.0(5.25) |  |  |
| 4 | 2.0(3.0) |  |  |  |  |  |
| **LN metastasis** |  |  |  |  |  |  |
| No |  |  | NA | 1.0(4.0) | No vs Yes | 0.88 |
| Yes | 2.0(3.0) |  |  | 1.0(2.5) |  |  |
| **ER** |  |  |  |  |  |  |
| Negative | 1.0(1.5) | -ve vs +ve | 0.40 | 1.0(1.0) | -ve vs +ve | 1.0 |
| Positive | 2.0(3.0) |  |  | 1.0(5.5) |  |  |
| **PR** |  |  |  |  |  |  |
| Negative | 1.0(0.0) | -ve vs +ve | 0.28 | 1.0(1.8) | -ve vs +ve | 0.67 |
| Positive | 2.0(3.0) |  |  | 1.0(4.0) |  |  |
| **HER2** |  |  |  |  |  |  |
| Negative | 2.0(3.0) | -ve vs +ve | 0.62 | 1.0(1.0) | -ve vs +ve | 0.71 |
| Positive | 1.0(3.0) |  |  | 1.0(5.5) |  |  |
| **Live status** |  |  |  |  |  |  |
| Alive | 2.0(3.01) | A vs D | 0.70 | 1.0(5.5) | A vs D | 1.0 |
| Dead | 1.55(3.0) |  |  | 1(1.0) |  |  |

^a^ Mann Whitney U testused forpairwise comparison. MBC; metastatic breast cancer, NMBC; non-metastatic breast cancer, IQR; interquartile range, LN; lymph node,ER; estrogen receptor, PR; progesterone receptor, HER2; human epidermal growth factor receptor2, DCIs; ductal carcinoma insitu, IDC; invasive ductal carcinoma, ILC; invasive lobular carcinoma, A; alive, D; dead. *Significance at p<0.05.

**Table S2-I.** Association of *UCA1* expression with clinicopathological characteristics of metastatic and non-metastatic breast cancer patients.

|  | **MBC** |  | **p^a^** | **NMBC** |  | **p^a^** |
| --- | --- | --- | --- | --- | --- | --- |
|  | **Median (IQR)** |  |  | **Median (IQR)** |  |  |
| **Age** |  |  |  |  |  |  |
| <50 | 1.25(2.08) | <50 vs ≥ 50 | 0.76 | 1.71(1.29) | <50 vs ≥ 50 | 0.28 |
| ≥ 50 | 0.28(1.82) |  |  | 1.14(1.74) |  |  |
| **Menopause** |  |  |  |  |  |  |
| Pre | 1.25(2.08) | Pre vs Post | 0.76 | 1.50(1.13) | Pre vs Post | 0.31 |
| Post | 0.28(1.82) |  |  | 0.99(1.97) |  |  |
| **Tumor Grade** |  |  |  |  |  |  |
| 1-2 | 1.83(2.84) | 1-2 vs 3 | 0.21 | 1.14(2.49) | 1-2 vs 3 | 0.87 |
| 3 | 0.22(1.11) |  |  | 1.55(0.54) |  |  |
| **Laterality** |  |  |  |  |  |  |
| Left | 0.13(1.35) | Left vs Right | **0.025*** | 1.51(2.11) | Left vs Right | 0.18 |
| Right | 1.85(4.56) |  |  | 0.87(1.11) |  |  |
| **Tumor size** |  |  |  |  |  |  |
| <2.5 | 1.85(0.99) | <2.5 vs ≥ 2.5 | 0.32 | 1.29(1.94) | <2.5 vs ≥ 2.5 | 0.67 |
| ≥ 2.5 | 0.24(1.68) |  |  | 1.39(0.96) |  |  |
| **Family History** |  |  |  |  |  |  |
| No | 0.73(1.90) | No vs Yes | 0.98 | 1.20(1.91) | No vs Yes | 0.49 |
| Yes | 0.97(2.15) |  |  | 1.71(0.66) |  |  |
| **Type** |  |  |  |  |  |  |
| DCIS | 2.44(11.1) | DCIS vs IDC | 0.12 | 1.83(1.27) | DCIS vs IDC | 0.47 |
| IDC | 0.24(1.85) | DCIS vs ILC | 0.53 | 1.29(1.93) | DCIS vs ILC | 1.0 |
| ILC | 1.34(0.09) | IDC vs ILC | 0.56 | 1.34(1.09) | IDC vs ILC | 0.61 |
| **Stage** |  |  |  |  |  |  |
| 3A |  |  | NA | 0.99(1.93) | 3A vs 3C | 0.087 |
| 3C |  |  |  | 1.75(3.70) |  |  |
| 4 | 0.73(2.04) |  |  |  |  |  |
| **LN metastasis** |  |  |  |  |  |  |
| No |  |  | NA | 1.30(1.15) | No vs Yes | 0.51 |
| Yes | 0.73(2.04) |  |  | 1.77(1.93) |  |  |
| **ER** |  |  |  |  |  |  |
| Negative | 0.22(1.08) | -ve vs +ve | 0.97 | 1.55(4.98) | -ve vs +ve | 0.40 |
| Positive | 1.17(1.99) |  |  | 1.21(1.77) |  |  |
| **PR** |  |  |  |  |  |  |
| Negative | 2.31(0.0) | -ve vs +ve | 0.35 | 2.18(1.51) | -ve vs +ve | 0.12 |
| Positive | 0.28(1.90) |  |  | 1.11(1.59) |  |  |
| **HER2** |  |  |  |  |  |  |
| Negative | 0.28(1.86) | -ve vs +ve | 0.25 | 0.99(1.35) | -ve vs +ve | 0.19 |
| Positive | 1.25(3.79) |  |  | 1.75(1.26) |  |  |
| **Livestatus** |  |  |  |  |  |  |
| Alive | 1.04(2.09) | A vs D | 0.74 | 1.30(1.41) | A vs D | 0.40 |
| Dead | 0.73(1.56) |  |  | 1.55(1.97) |  |  |

^a^ Mann Whitney U testused forpairwise comparison. MBC; metastatic breast cancer, NMBC; non-metastatic breast cancer, IQR; interquartile range, LN; lymph node, ER; estrogen receptor, PR; progesterone receptor, HER2; human epidermal growth factor receptor2, DCIs; ductal carcinoma insitu, IDC; invasive ductal carcinoma, ILC; invasive lobular carcinoma, A; alive, D; dead. *Significance at p<0.05.

**Table S2-J.** Association of *SPRY4T1* expression with clinicopathological characteristics of metastatic and non-metastatic breast cancer patients.

| **Features** | **MBC** |  | **p^a^** | **NMBC** |  | **p^a^** |
| --- | --- | --- | --- | --- | --- | --- |
|  | **Median (IQR)** |  |  | **Median (IQR)** |  |  |
| **Age** |  |  |  |  |  |  |
| <50 | 1.07(0.24) | <50 vs ≥ 50 | 0.42 | 1.88(49.6) | <50 vs ≥ 50 | 0.12 |
| ≥ 50 | 1.09(0.82) |  |  | 1.13(1.13) |  |  |
| **Menopause** |  |  |  |  |  |  |
| Pre | 1.07(0.24) | Pre vs Post | 0.42 | 1.81(41.0) | Pre vs Post | 0.34 |
| Post | 1.09(0.82) |  |  | 1.13(0.97) |  |  |
| **Tumor Grade** |  |  |  |  |  |  |
| 1-2 | 1.09(0.25) | 1-2 vs 3 | 0.74 | 1.53(4.81) | 1-2 vs 3 | 0.71 |
| 3 | 1.09(18.0) |  |  | 1.32(25.8) |  |  |
| **Laterality** |  |  |  |  |  |  |
| Left | 1.53(26.9) | Left vs Right | **0.027*** | 1.22(15.2) | Left vs Right | 0.53 |
| Right | 1.07(0.23) |  |  | 1.88(0.81) |  |  |
| **Tumor size** |  |  |  |  |  |  |
| <2.5 | 1.17(0.34) | <2.5 vs ≥ 2.5 | 0.33 | 1.32(0.99) | <2.5 vs ≥ 2.5 | 1.0 |
| ≥ 2.5 | 1.07(0.67) |  |  | 8.54(15.0) |  |  |
| **Family History** |  |  |  |  |  |  |
| No | 1.08(0.30) | No vs Yes | 0.43 | 1.60(11.5) | No vs Yes | 0.48 |
| Yes | 1.26(0.53) |  |  | 1.13(0.65) |  |  |
| **Type** |  |  |  |  |  |  |
| DCIS | 1.43(1.24) | DCIS vs IDC | 0.52 | 1.42(0.32) | DCIS vs IDC | 1.0 |
| IDC | 1.1(0.26) | DCIS vs ILC | 0.53 | 1.18(1.34) | DCIS vs ILC | 0.67 |
| ILC | 1.01(0.063) | IDC vs ILC | 0.41 | 1.58(0.46) | IDC vs ILC | 0.79 |
| **Stage** |  |  |  |  |  |  |
| 3A |  |  |  | 1.18(1.08) | 3A vs 3C | 0.058 |
| 3C |  |  |  | 51.5(40.7) |  |  |
| 4 | 1.09(0.31) |  |  |  |  |  |
| **LN metastasis** |  |  |  |  |  |  |
| No |  |  |  | 1.32(0.93) | No vs Yes | 0.56 |
| Yes | 1.09(0.31) |  |  | 1.61(50.1) |  |  |
| **ER** |  |  |  |  |  |  |
| Negative | 1.06(0.15) | -ve vs +ve | 0.30 | 50.9(53.1) | -ve vs +ve | 0.10 |
| Positive | 1.09(0.67) |  |  | 1.15(0.92) |  |  |
| **PR** |  |  |  |  |  |  |
| Negative | 0.80(0.0) | -ve vs +ve | 0.32 | 28.1(54.4) | -ve vs +ve | 0.19 |
| Positive | 1.09(0.46) |  |  | 1.18(1.16) |  |  |
| **HER2** |  |  |  |  |  |  |
| Negative | 1.15(0.81) | -ve vs +ve | **0.025*** | 1.13(1.08) | -ve vs +ve | 0.13 |
| Positive | 1.06(0.39) |  |  | 1.68(50.6) |  |  |
| **Livestatus** |  |  |  |  |  |  |
| Alive | 1.14(0.25) | A vs D | 0.34 | 1.22(0.80) | A vs D | 0.34 |
| Dead | 1.07(0.99) |  |  | 2.04(49.8) |  |  |

^a^ Mann Whitney U testused forpairwise comparison. MBC; metastatic breast cancer, NMBC; non-metastatic breast cancer, IQR; interquartile range, LN; lymph node,ER; estrogen receptor, PR; progesterone receptor, HER2; human epidermal growth factor receptor2, DCIs; ductal carcinoma insitu, IDC; invasive ductal carcinoma, ILC; invasive lobular carcinoma, A; alive, D; dead. *Significance at p<0.05.

**Table S2-K.** Association of *AK058003* expression with clinicopathological characteristics of metastatic and non-metastatic breast cancer patients.

|  | **MBC** |  | **p^a^** | **NMBC** |  | **p^a^** |
| --- | --- | --- | --- | --- | --- | --- |
|  | **Median (IQR)** |  |  | **Median (IQR)** |  |  |
| **Age** |  |  |  |  |  |  |
| <50 | 2.75(8.8) | <50 vs ≥ 50 | 0.15 | 8.69(15.1) | <50 vs ≥ 50 | **0.005*** |
| ≥ 50 | 1.43(4.12) |  |  | 2.0(2.76) |  |  |
| **Menopause** |  |  |  |  |  |  |
| Pre | 2.75(8.8) | Pre vs Post | 0.15 | 7.02(13.7) | Pre vs Post | **0.024*** |
| Post | 1.43(4.12) |  |  | 2.0(2.89) |  |  |
| **Tumor Grade** |  |  |  |  |  |  |
| 1-2 | 2.43(8.09) | 1-2 vs 3 | 0.83 | 2.5(3.12) | 1-2 vs 3 | 0.10 |
| 3 | 2.36(3.62) |  |  | 8.69(14.7) |  |  |
| **Laterality** |  |  |  |  |  |  |
| Left | 2.40(3.40) | Left vs Right | 0.93 | 2.96(5.0) | Left vs Right | 0.62 |
| Right | 3.29(8.48) |  |  | 2.3(5.91) |  |  |
| **Tumor size** |  |  |  |  |  |  |
| <2.5 | 6.97(6.68) | <2.5 vs ≥ 2.5 | **0.017*** | 4.17(8.53) | <2.5 vs ≥ 2.5 | 0.22 |
| ≥ 2.5 | 1.44(2.72) |  |  | 2.35(1.27) |  |  |
| **Family History** |  |  |  |  |  |  |
| No | 2.20(4.39) | No vs Yes | 0.59 | 2.65(6.50) | No vs Yes | 0.63 |
| Yes | 3.48(7.3) |  |  | 2.91(1.48) |  |  |
| **Type** |  |  |  |  |  |  |
| DCIS | 2.35(3.63) | DCIS vs IDC | 0.92 | 10.5(7.60) | DCIS vs IDC | 0.44 |
| IDC | 2.20(4.24) | DCIS vs ILC | 0.13 | 3.01(3.35) | DCIS vs ILC | 0.33 |
| ILC | 11.5(0.88) | IDC vs ILC | 0.095 | 0.002(0.00005) | IDC vs ILC | **0.048*** |
| **Stage** |  |  |  |  |  |  |
| 3A |  |  |  | 2.69(2.94) | 3A vs 3C | 0.12 |
| 3C |  |  |  | 10.3(12.8) |  |  |
| 4 | 2.40(5.0) |  |  |  |  |  |
| **LN metastasis** |  |  |  |  |  |  |
| No |  |  |  | 2.30(1.82) | No vs Yes | 0.29 |
| Yes | 2.4(5.0) |  |  | 5.04(6.06) |  |  |
| **ER** |  |  |  |  |  |  |
| Negative | 4.72(5.01) | -ve vs +ve | 0.28 | 2.0(10.4) | -ve vs +ve | 0.94 |
| Positive | 2.04(4.53) |  |  | 2.96(3.13) |  |  |
| **PR** |  |  |  |  |  |  |
| Negative | 12.4(0.0) | -ve vs +ve | 0.19 | 15.0(16.6) | -ve vs +ve | 0.074 |
| Positive | 2.36(4.25) |  |  | 2.69(3.04) |  |  |
| **HER2** |  |  |  |  |  |  |
| Negative | 2.43(3.94) | -ve vs +ve | 0.69 | 2.30(1.42) | -ve vs +ve | 0.11 |
| Positive | 2.36(7.53) |  |  | 6.71(13.9) |  |  |
| **Live status** |  |  |  |  |  |  |
| Alive | 3.92(7.26) | A vs D | 0.24 | 2.80(8.24) | A vs D | 0.66 |
| Dead | 1.44(4.33) |  |  | 4.17(3.44) |  |  |

^a^ Mann Whitney U testused forpairwise comparison. MBC; metastatic breast cancer, NMBC; non-metastatic breast cancer, IQR; interquartile range, LN; lymph node, ER; estrogen receptor, PR; progesterone receptor, HER2; human epidermal growth factor receptor2, DCIs; ductal carcinoma insitu, IDC; invasive ductal carcinoma, ILC; invasive lobular carcinoma, A; alive, D; dead. *Significance at p<0.05.

**Table S2-L.** Association of *MALAT1* expression with clinicopathological characteristics of metastatic and non-metastatic breast cancer patients.

| **Features** | **MBC** |  | **p^a^** | **NMBC** |  | **p^a^** |
| --- | --- | --- | --- | --- | --- | --- |
|  | **Median (IQR)** |  |  | **Median (IQR)** |  |  |
| **Age** |  |  |  |  |  |  |
| <50 | 1.24(3.32) | <50 vs ≥ 50 | 0.86 | 2.08(1.37) | <50 vs ≥ 50 | 1.0 |
| ≥ 50 | 1.51(2.36) |  |  | 1.55(5.81) |  |  |
| **Menopause** |  |  |  |  |  |  |
| Pre | 1.24(3.32) | Pre vs Post | 0.86 | 2.10(1.49) | Pre vs Post | 0.60 |
| Post | 1.51(2.36) |  |  | 1.0(2.67) |  |  |
| **Tumor Grade** |  |  |  |  |  |  |
| 1-2 | 2.16(3.45) | 1-2 vs 3 | 0.12 | 1.72(3.43) | 1-2 vs 3 | 0.64 |
| 3 | 0.74(1.26) |  |  | 2.08(1.81) |  |  |
| **Laterality** |  |  |  |  |  |  |
| Left | 0.91(1.47) | Left vs Right | 0.06 | 1.72(3.16) | Left vs Right | 0.92 |
| Right | 2.69(3.97) |  |  | 2.08(2.17) |  |  |
| **Tumor size** |  |  |  |  |  |  |
| <2.5 | 1.66(3.02) | <2.5 vs ≥ 2.5 | 0.90 | 2.10(1.88) | <2.5 vs ≥ 2.5 | 0.37 |
| ≥ 2.5 | 1.37(2.32) |  |  | 0.34(15.0) |  |  |
| **Family History** |  |  |  |  |  |  |
| No | 1.16(2.19) | No vs Yes | 0.34 | 1.72(2.27) | No vs Yes | 0.55 |
| Yes | 2.53(3.74) |  |  | 2.11(6.61) |  |  |
| **Type** |  |  |  |  |  |  |
| DCIS | 1.18(16.2) | DCIS vs IDC | 0.71 | 29.6(27.4) | DCIS vs IDC | 0.19 |
| IDC | 1.20(2.06) | DCIS vs ILC | 0.53 | 1.35(2.26) | DCIS vs ILC | 0.67 |
| ILC | 8.50(3.29) | IDC vs ILC | **0.029*** | 1.92(0.92) | IDC vs ILC | 0.59 |
| **Stage** |  |  |  |  |  |  |
| 3A | - |  |  | 2.10(2.34) | 3A vs 3C | 0.53 |
| 3C | - |  |  | 1.72(1.77) |  |  |
| 4 | 1.37(2.32) |  |  |  |  |  |
| **LN metastasis** |  |  |  |  |  |  |
| No |  |  |  | 1.0(0.84) | No vs Yes | 0.85 |
| Yes | 1.24(2.27) |  |  | 2.09(1.04) |  |  |
| **ER** |  |  |  |  |  |  |
| Negative | 2.01(1.08) | -ve vs +ve | 0.89 | 1.35(1.98) | -ve vs +ve | 0.58 |
| Positive | 1.24(2.53) |  |  | 2.09(2.23) |  |  |
| **PR** |  |  |  |  |  |  |
| Negative | 2.46(0.0) | -ve vs +ve | 0.71 | 2.30(14.5) | -ve vs +ve | 0.66 |
| Positive | 1.24(2.45) |  |  | 1.35(2.28) |  |  |
| **HER2** |  |  |  |  |  |  |
| Negative | 1.16(2.34) | -ve vs +ve | 0.43 | 0.97(1.94) | -ve vs +ve | 0.25 |
| Positive | 2.46(2.32) |  |  | 2.30(1.26) |  |  |
| **Livestatus** |  |  |  |  |  |  |
| Alive | 1.09(1.78) | A vs D | 0.19 | 0.98(2.13) | A vs D | 0.41 |
| Dead | 2.09(2.82) |  |  | 2.10(1.50) |  |  |

^a^ Mann Whitney U testused forpairwise comparison. MBC; metastatic breast cancer, NMBC; non-metastatic breast cancer, IQR; interquartile range, LN; lymph node, ER; estrogen receptor, PR; progesterone receptor, HER2; human epidermal growth factor receptor2, DCIs; ductal carcinoma insitu, IDC; invasive ductal carcinoma, ILC; invasive lobular carcinoma, A; alive, D; dead. *Significance at p<0.05.
